# Days alive and out of hospital for adult female and male cardiac surgery patients: a population-based cohort study

**DOI:** 10.1186/s12872-024-03862-7

**Published:** 2024-04-20

**Authors:** Angela Jerath, Christopher J. D. Wallis, Stephen Fremes, Vivek Rao, Terrence M. Yau, Kiyan Heybati, Douglas S. Lee, Harindra C. Wijeysundera, Jason Sutherland, Peter C. Austin, Duminda N. Wijeysundera, Dennis T. Ko

**Affiliations:** 1 Department of Anesthesia, Sunnybrook Health Sciences Center, Toronto, ON Canada; 2 Department of Anesthesiology and Pain Medicine, University of Toronto, Toronto, ON Canada; 3 ICES, 2075 Bayview Avenue, Toronto, ON Canada; 4 Schulich Heart Centre, Sunnybrook Research Institute, Sunnybrook Health Sciences Center, Toronto, ON Canada; 5 Division of Urology, Department of Surgery, University of Toronto, Toronto, ON Canada; 6 Division of Urology, Department of Surgery, Mount Sinai Hospital, Toronto, ON Canada; 7 Department of Surgical Oncology, University Health Network, Toronto, ON Canada; 8 Division of Cardiovascular Surgery, Sunnybrook Health Sciences Center, Toronto, ON Canada; 9 Division of Cardiovascular Surgery, Toronto General Hospital-University Health Network, Toronto, ON Canada; 10 Toronto General Hospital Research Institute, Toronto, ON Canada; 11 Division of Cardiovascular Surgery, University of Toronto, Toronto, ON Canada; 12 Mayo Clinic Alix School of Medicine, Mayo Clinic, Rochester, MN USA; 13 Division of Cardiology, Toronto General Hospital-University Health Network, Toronto, ON Canada; 14 Centre for Health Services and Policy Research, University of British Columbia, Vancouver, BC Canada; 15 Department of Anesthesia, St. Michael’s Hospital, Toronto, ON Canada

**Keywords:** Days at home, Cardiac surgery, Sex, Epidemiology, Outcomes, Validation

## Abstract

**Background:**

Research shows women experience higher mortality than men after cardiac surgery but information on sex-differences during postoperative recovery is limited. Days alive and out of hospital (DAH) combines death, readmission and length of stay, and may better quantify sex-differences during recovery. This main objective is to evaluate (i) how DAH at 30-days varies between sex and surgical procedure, (ii) DAH responsiveness to patient and surgical complexity, and (iii) longer-term prognostic value of DAH.

**Methods:**

We evaluated 111,430 patients (26% female) who underwent one of three types of cardiac surgery (isolated coronary artery bypass [CABG], isolated non-CABG, combination procedures) between 2009 – 2019. Primary outcome was DAH at 30 days (DAH_30_), secondary outcomes were DAH at 90 days (DAH_90_) and 180 days (DAH_180_). Data were stratified by sex and surgical group. Unadjusted and risk-adjusted analyses were conducted to determine the association of DAH with patient-, surgery-, and hospital-level characteristics. Patients were divided into two groups (below and above the 10th percentile) based on the number of days at DAH_30._ Proportion of patients below the 10th percentile at DAH_30_ that remained in this group at DAH_90_ and DAH_180_ were determined.

**Results:**

DAH_30_ were lower for women compared to men (22 vs. 23 days), and seen across all surgical groups (isolated CABG 23 vs. 24, isolated non-CABG 22 vs. 23, combined surgeries 19 vs. 21 days). Clinical risk factors including multimorbidity, socioeconomic status and surgical complexity were associated with lower DAH_30_ values, but women showed lower values of DAH_30_ compared to men for many factors. Among patients in the lowest 10th percentile at DAH_30_, 80% of both females and males remained in the lowest 10th percentile at 90 days, while 72% of females and 76% males remained in that percentile at 180 days.

**Conclusion:**

DAH is a responsive outcome to differences in patient and surgical risk factors. Further research is needed to identify new care pathways to reduce disparities in outcomes between male and female patients.

**Supplementary Information:**

The online version contains supplementary material available at 10.1186/s12872-024-03862-7.

## Introduction

Sex-differences in outcomes after cardiac surgery have been identified in early studies with poorer recovery demonstrated in women compared to men [1–3]. Overall mortality after cardiac surgery remains low (1–4%) [4] but this endpoint fails to capture the high incidence (16–35%) [5–7] of new morbidity after surgery that slows patient recovery. Further study of outcomes after cardiac surgery and how this differs between women and men is essential with more elderly multimorbid patients undergoing high risk procedures in contemporary practice that places them at elevated risk of new complications after surgery [8]. A composite outcome called failure to achieve an uneventful recovery that is based on complications has identified specific perioperative risk factors including female sex that are associated with higher risk of postoperative adverse events [9, 10]. Days alive and out of hospital (DAH) is a novel outcome that has been validated and used in non-cardiac surgery to capture global outcomes of the patient experience [11, 12]. DAH is a composite endpoint that integrates death, duration of hospitalization and long-term care, and can be measured over any time period. Non-cardiac surgery studies have shown this metric is appropriately responsive to the patients’ preoperative health status and new problems that arise from the surgical experience. In addition, noncardiac surgery studies indicate that early assessment of DAH (i.e., within 30-days after surgery) has useful prognostic qualities for understanding the longer-term trajectory of patients’ recovery [11]. DAH has become increasingly used as a global metric of patient outcomes for cardiology trials [13, 14], recommended measurement endpoint for perioperative studies, and used to evaluate trends in cardiac disease and policy implications [15–17].

DAH has not been evaluated in cardiac surgery, nor has the effect of patient sex or type of surgery on DAH. This knowledge gap forms the basis of this population cohort study which has 3 objectives. First, to determine how DAH varies between patient sex and cardiac surgical procedure. Second, to evaluate the construct validity of DAH based on its association with patient (e.g., sex), surgical (e.g., type) and hospital factors. Third, to determine if the early DAH value has longer-term prognostic value in female and male cardiac surgical patients.

## Methods

### Settings and data sources

This study is reported according to the Strengthening the Reporting of Observational Studies in Epidemiology (STROBE) guidelines and the Reporting of Studies Conducted Using Observational Routinely Collected Health Data (RECORD) statement. We conducted a retrospective cohort study using population-based administrative healthcare databases in Ontario, Canada. The use of data in this project was authorized under Sect. 45 of Ontario’s Personal Health Information Protection Act, which does not require review by a research ethics board. We used CorHealth Ontario to identify cardiac surgeries and specific cardiac health information. The Registered Persons Database (RPDB), Vital Statistics and Ontario census data were used to extract demographics, socioeconomic status and mortality. The Canadian Institute of Health Information Discharge Abstract Database (CIHI-DAD) captures all acute care hospital admissions and provided information additional information during primary hospitalization, and any readmissions. We used the Ontario Health Insurance Plan (OHIP) database to capture all physician service claim data. The Continuing Care Reporting System (CCRS) database was used to capture patients needing long-term care support. Specialized databases (Ontario Diabetes Database, Asthma Database, Chronic Obstructive Pulmonary Disease Database, Ontario Hypertension Database) were used to identify specific comorbidities. Laboratory values were obtained from the Ontario Laboratory Information System (OLIS). Data were linked through unique anonymized patient identifier numbers. Key variables and codes used are summarized in Supplemental Digital Content Tables S1 [11, 18, 19].

### Study cohort

We identified adults (≥ 18 years) patients who underwent common elective and emergency cardiac surgeries between 2009 – 2019 in Ontario hospitals. The procedures included coronary bypass surgery (CABG), aneurysectomy, valve repair/replacement, and aortic surgery. We excluded less commonly performed surgeries such as adult congenital heart procedures, heart transplantation and ventricular assist device implantation. The surgical procedures were placed into 3 clinically sensible groups based on operative complexity and commonly described description: (i) isolated CABG, (ii) single non-CABG procedures (e.g., single valve repair/replacement), and (iii) combined (2 or more) procedures (e.g., valve and CABG surgery, multiple valve surgery, aortic and CABG/valve surgery) [5, 7]. We excluded intraoperative deaths (*n* = 219) in order to assess DAH in the postoperative period, patients with missing unique identifier number or death date (*n* = 75), patient procedure dates and institution identified in DAD but not verified in CorHealth data (*n* = 5,350). For patients with multiple surgeries during the study period, we excluded all but the first procedure (*n* = 5,673).

### Outcome

The primary outcome was DAH at 30 days after surgery (referred to as DAH_30_). This was calculated using mortality, hospital length of stay, and readmissions between the date of the index surgery and the 30th postoperative day using validated sources from CIHI-DAD (supplemental figure S1) [20]. The approach to calculating DAH has been previously described. In brief, the duration a patient stays in hospital is subtracted from the measured time frame, for example, a patient who survived and was discharged 20 days after the indexed surgery had a DAH_30_ of 10 days. Patients who died at any time during this 30-day period were assigned a DAH_30_ of 0 days. The secondary outcomes were DAH at 90 days (DAH_90_) and 180 days (DAH_180_), which were determined using similar calculations.

### Covariates

Demographics (age, sex) were identified from the RPDB. Comorbidities (coronary artery disease, diabetes, hypertension, chronic obstructive pulmonary disease, atrial fibrillation, asthma, body mass index, stroke, chronic liver disease, smoking status, anemia, left ventricular ejection fraction, components of the Charlson comorbidity index score) were extracted from CorHealth, OLIS, CIHI-DAD (using ICD-10 codes from hospital admissions) and specialized validated Ontario databases within 3 years before the index surgery [21–24]. Severity of preoperative kidney dysfunction was classified into one of 5 stages of KDIGO renal function based on the patient’s estimated glomerular function prior to surgery [25]. To capture level of patient acuity and sickness before surgery, we recorded those patients needing preoperative intensive level care and DAH in the 3 months preceding the day of indexed surgery. Frailty was estimated using the hospital frailty risk score [26]. Socioeconomic status was based on neighborhood income quintile (1 is lowest, 5 is highest) and extracted from StatsCan. Major (grade 3–4 Clavien-Dindo) complications within 30-days after surgery needing ICU readmission, reoperation, rehospitalization, or another advanced intervention (e.g., pacemaker, angioplasty, dialysis, tracheostomy, prolonged ventilation, prolonged ICU admission, balloon pump, endoscopic procedure, extra-corporeal support) were extracted from CorHealth, CIHI-DAD, OLIS and OHIP [27–29]. Surgery variables include procedure type, duration, and urgency. Hospital variables included surgical volume, teaching status and bed number [20].

### Statistical analysis

Descriptive statistics were used to initially compare male and female patients using frequency (proportion) for categorical variables, median (interquartile range, IQR) for continuous variables and standardized difference. Subsequently, men and women were studied and reported separately. For each sex-group, descriptive statistics were estimated for each of the three surgical groups (single CABG, single non-CABG, combined surgeries). Construct validity describes how DAH responds to patient and surgical risk factors. We expect DAH to show convergent validity with lower DAH values in patients undergoing more complex procedures or display higher burden of chronic diseases. Hence, to evaluate construct validity of patient level factors and type of surgery, and how this differs for female and male patients, we summarized the unadjusted effect of common patient comorbidities (e.g., diabetes, atrial fibrillation, stroke, body mass index, chronic obstructive pulmonary disease, chronic kidney and liver disease, smoking status, socioeconomic status, and surgery type) on DAH at 30-days using median (IQR). To study the adjusted association of patient, surgery and hospital factors with DAH, we used a multivariable median regression model to model the association of covariates (with the median DAH [30]. This approach has been previously used to manage the skewed nature of our data [9, 16]. The model incorporated hospital-specific random effects to account for within-hospital clustering. Separate models were developed for male and female patients for DAH at 30, 90 and 180 days using the above covariates. A sensitivity analysis was performed after removal of complications from the model. To formally evaluate whether there was an interaction between patient sex and modifiable risk factors (e.g., hospital teaching status, procedure group), we fit the above model to the entire sample of men and women combined and included an interaction term between patient sex and the given risk factor. This was done sequentially for one risk factor at a time. Risk adjusted models were performed on a cohort of 87,826 patients during the study time frame after removal of missing variables (rural 0.1%, income quintile 0.3%, surgery duration 0.2%, left ventricular function 3%, body mass index 5%, smoking status 2%, kidney function 14%). No imputation of data was performed.

We explored the prognostic implications of our sickest patients with the fewest number of DAH at 30 days. After removing patients who died during this 30-day period, patients were ranked based on their value of DAH at 30 days, and then placed into 2 groups—those in the lowest 10th percentile and those above this percentile. The 10th percentile cut-off has been previously used for non-cardiac surgery to capture those patients with the poorest number of DAH, and is appropriate given the left skewness of the data distribution [11]. Patient, surgical and hospital characteristics of those below and above the 10th percentile were quantified using median (IQR), frequency (percentage) and standardized differences. We subsequently determined the proportion of patients in below and above the 10th percentile at 30 days that remained within these group at 90 and 180 days.

The trajectory and morbidity of individual cardiac surgeries subtly vary. For example, in the single non-CABG group, outcomes may be different for men and women undergoing mitral valve versus tricuspid valve surgery. To further explore this, we performed pre-specified sub-group analyses within the isolated non-CABG and combined procedure surgical groups to study these differences in operations between patient sex. This was performed using the same above risk adjusted model for the outcomes of DAH at 30 days.

All analyses were conducted using Microsoft Excel (v.2010, Redmond, WA), SAS version 9.4 (SAS Institute, Cary, US) and R statistical software [31–33]. Two-sided *p*-values < 0.05 were considered statistically significant. No statistical power calculation was performed prior to conducting this study and the sample size was based on the available data meeting the above eligibility requirements. This sample was based on our previous experience in conducting health services research using this patient population and research design [19, 34].

## Results

The cohort included 111,430 patients with 28,437 (26%) women and 82,993 (74%) men. The majority (63%) of procedures were isolated CABG, followed by combined (21%) and single non-CABG (16%) surgeries (Table 1). Overall, patients undergoing combined surgeries were older with more chronic medical diseases compared to isolated CABG or isolated non-CABG surgery with fewer DAH at all time points compared to isolated CABG and non-CABG surgery.

**Table 1:** Descriptive characteristics of cardiac surgery patients stratified by sex and surgical group

	**Female** ***N* = 28,437**	**Male** ***N* = 82,993**	**Total** ***N* = 111,430**	**Standardized Difference**
**Patient factors**				
Age	69 (61–76)	66 (59–74)	67 (59–74)	0.24
Atrial fibrillation	4,157 (14.6%)	8,934 (10.8%)	13,091 (11.7%)	0.12
Anemia	2,999 (10.5%)	4,806 (5.8%)	7,805 (7.0%)	0.17
Asthma	5,323 (18.7%)	8,770 (10.6%)	14,093 (12.6%)	0.23
CAD	14,351 (50.5%)	46,061 (55.5%)	60,412 (54.2%)	0.1
Stroke	806 (2.8%)	2,090 (2.5%)	2,896 (2.6%)	0.02
Dementia	83 (0.3%)	181 (0.2%)	264 (0.2%)	0.01
Diabetes	12,391 (43.6%)	34,881 (42.0%)	47,272 (42.4%)	0.03
Dialysis	475 (1.7%)	1,299 (1.6%)	1,774 (1.6%)	0.01
Hypertension	23,961 (84.3%)	68,458 (82.5%)	92,419 (82.9%)	0.05
Chronic liver disease	216 (0.8%)	617 (0.7%)	833 (0.7%)	0
Myocardial infarction	7,147 (25.1%)	23,326 (28.1%)	30,473 (27.3%)	0.07
PVD	1,858 (6.5%)	5,253 (6.3%)	7,111 (6.4%)	0.01
Primary cancer	661 (2.3%)	2,118 (2.6%)	2,779 (2.5%)	0.01
Secondary cancer	109 (0.4%)	246 (0.3%)	355 (0.3%)	0.01
COPD	3,456 (12.2%)	8,168 (9.8%)	11,624 (10.4%)	0.07
^b^Chronic kidney disease				
Stage 1	6,149 (21.6%)	22,947 (27.6%)	29,096 (26.1%)	0.14
Stage 2	10.947 (38.5%)	32,419 (39.1%)	43,366 (38.9%)	0.01
Stage 3	6,249 (22.0%)	13,286 (16.0%)	19,535 (17.5%)	0.15
Stage 4	1,035 (3.6%)	1,991 (2.4%)	3,026 (2.7%)	0.07
Stage 5	344 (1.2%)	903 (1.1%)	1,247 (1.1%)	0.01
CCI ≥ 2	9,027 (31.7%)	23,775 (28.6%)	32,802 (29.4%)	0.07
Rural	4,237 (14.9%)	12,539 (15.1%)	16,776 (15.1%)	0.01
Income quintile				
Q1	6,284 (22.2%)	14,752 (17.8%)	21,036 (18.9%)	0.11
Q2	6,052 (21.4%)	16,582 (20.0%)	22,634 (20.4%)	0.03
Q3	5,677 (20.0%)	17,057 (20.6%)	22,734 (20.5%)	0.01
Q4	5,268 (18.6%)	17,156 (20.7%)	22,424 (20.2%)	0.05
Q5	5,055 (17.8%)	17,162 (20.7%)	22,217 (20.0%)	0.07
Body mass index				
≤ 25	8,206 (28.9%)	19,187 (23.1%)	27,393 (24.6%)	0.13
26–30	8,574 (30.2%)	32,880 (39.6%)	41,454 (37.2%)	0.2
≥ 31	10,274 (36.1%)	26,736 (32.2%)	37,010 (33.2%)	0.08
LVEF				
< 20%	241 (0.8%)	1,509 (1.8%)	1,750 (1.6%)	0.08
20%—34%	1,715 (6.0%)	7,190 (8.7%)	8,905 (8.0%)	0.1
35%—49%	4,378 (15.4%)	17,549 (21.1%)	21,927 (19.7%)	0.15
≥ 50%	21,233 (74.7%)	54,069 (65.1%)	75,302 (67.6%)	0.21
Smoking status				
Current	4,523 (16.1%)	16,682 (20.3%)	21,205 (19.2%)	0.11
Former	6,799 (24.1%)	31,231 (38.0%)	38,030 (34.5%)	0.3
Never	16,554 (58.8%)	33,472 (40.7%)	50,026 (45.3%)	0.37
Frailty	2 (0–5)	1 (0–3)	1 (0–4)	0.19
Preop DAH (3m—1d)	89 (82–92)	89 (83–92)	89 (83–92)	0.04
**Surgery**				
Surgery duration (min)	268 (223–323)	273 (230–326)	271 (228–325)	0.07
Re-do surgery	833 (5.5%)	1,835 (4.4%)	2,668 (4.7%)	0.05
Preop ICU level care	15,913 (56%)	47,358 (57.1%)	63,271 (56.8%)	0.02
Elective	17,975 (63.2%)	51,316 (61.8%)	69,291 (62.2%)	0.03
Urgent/Emergent	10,462 (36.8%)	31,677 (38.2%)	42, 139 (37.8%)	0.03
Surgical group				
Combined surgeries	7,393 (26.0%)	16,352 (19.7%)	23,745 (21.3%)	0.15
Isolated non-CABG procedure	6,933 (24.4%)	10,298 (12.4%)	17,231 (15.5%)	0.31
Isolated CABG	14,111 (49.6%)	56,343 (67.9%)	70,454 (63.2%)	0.38
**Hospital**				
Teaching hospital	17,634 (62.0%)	50,531 (60.9%)	68,165 (61.2%)	0.02
ICU Beds	61 (35–77)	60 (35–74)	60 (35–74)	0.03
Surgical Beds	131 (84–210)	131 (84–176)	131 (84–176)	0.01
Total Beds	367 (288–514)	367 (291–513)	367 (291–513)	0
**Outcomes**				
30-day mortality	418 (1.5%)	778 (0.9%)	1,196 (1.1%)	0.05
90-day mortality	670 (2.4%)	1,238 (1.5%)	1,908 (1.7%)	0.06
ICU LOS (h)	47 (25–95)	37 (23–75)	42 (24–78)	0.17
ALC patient	1,380 (4.9%)	2,155 (2.6%)	3,535 (3.2%)	0.12
Postop LOS (d)	7 (6–10)	6 (5–8)	6 (5–9)	0.38
Major complications	6,148 (21.6%)	14,132 (17.0%)	20,280 (18.2%)	0.12
30-day hospital readmission	3840 (13.5%)	8293 (10.0%)	12,133 (10.9%)	0.11
90-day hospital readmission	5681 (20%)	12,357 (14.9%)	18,038 (16.2%)	0.13
180-day hospital readmission	6998 (24.6%)	15,697 (18.9%)	22,695 (20.4%)	0.14
^a^DAH_30_	22 (16–24)	23 (20–25)	23 (19–25)	0.42
	18.5 ± 7.8	20.8 ± 6.7	20.2 ± 7.1	0.31
^a^DAH_90_	81 (74–84)	83 (79–85)	83 (78–85)	0.42
	73.0 ± 20.7	77.5 ± 16.8	76.3 ± 18.0	0.24
^a^DAH_180_	171 (162–173)	173 (168–175)	172 (166–174)	0.41
	156.5 ± 39.8	163.4 ± 32.0	161.6 ± 34.2	0.19

*ALC alternate care; CAD coronary artery disease; CCI Charlson comorbidity index; COPD chronic obstructive pulmonary disease; d days; DAH days alive and out of hospital; h hours; ICU intensive care unit; LOS length of stay; LVEF left ventricular ejection fraction; m month; PVD peripheral vascular disease*

^*a*^*DAH described using median (inter-quartile range) and mean* ± *standard deviation*

^*b*^*Chronic kidney disease divided into 5 groups using Kidney Disease Improving Global Outcomes (KDIGO) classification based on preoperative estimated glomerular filtration rate*

Compared to male patients, female patients were more advanced in age (69y vs. 66y), showed greater burden of comorbidities, a higher proportion of lower socioeconomic status (income quintile 1/2, 43% vs. 37%) and underwent more complex combined surgeries (25% vs. 19%), Table 1. The median DAH_30_ for men and women was 23 and women 22 days respectively (*p* < 0.001), Table 1. Among male patients, DAH_30_ for isolated CABG, isolated non-CABG and combined surgery were 24, 23 and 21 days respectively, Supplemental Table S2. Number of days at DAH_30_ in women undergoing for isolated CABG, isolated non-CABG and combined surgery were lower than men at 23, 22 and 19 days respectively. Similar trends with fewer DAH among women were sustained at 90 and 180 days, Supplemental Table S2.

The unadjusted association of patient factors with DAH is summarized in Table 2. Presence of common comorbidities were associated with a greater reduction in median DAH_30_ among women compared to men for e.g., Charlson Comorbidity Index ≥ 2: 19 vs. 22 days, grade 5 kidney disease: 13 vs. 17 days. The number of DAH_30_ fell in patients of lower socioeconomic status. For each income quintile group women showed fewer DAH compared to men (median DAH_30_ income quintile 1 to 5, 21–22 [female] vs. 23–24 [male]).

**Table 2:** Unadjusted changes in days alive and out of hospital at 30- and 90-days for patient factors stratified by patient sex

	**DAH**_**30**_			
	**Female**		**Male**	
	**Median**	**IQR**	**Median**	**IQR**
**Patient factors**				
CCI ≥ 2	19	10–23	22	16–24
COPD	19	11–23	21	14–24
Diabetes	21	15–24	23	19–25
Peripheral vascular disease	20	12–23	22	16–24
Atrial fibrillation	18	9–22	20	13–23
Stroke/TIA	18	7–23	21	13–24
Chronic liver disease	13	0–21	18	6–23
Chronic kidney disease				
Stage 1	23	21–25	24	22–25
Stage 2	22	18–24	23	21–25
Stage 3	19	11–23	21	15–24
Stage 4	13	0–20	16	1–22
Stage 5	13	0–20	17	3–22
Income Quintile				
Q1	21	14–24	23	19–25
Q2	22	16–24	23	20–25
Q3	22	16–24	24	20–25
Q4	22	16–24	24	20–25
Q5	22	17–24	24	21–25
Smoking Status				
Current	22	16–24	24	20–25
Ex-smoker	22	16–24	23	20–25
Never smoked	22	16–24	23	20–25
Left ventricular ejection fraction				
< 20%	19	4–23	20	10–24
20%—34%	19	9–23	22	16–24
35%—49%	21	14–24	23	19–25
≥ 50%	22	17–24	24	21–25
Preoperative ICU	22	16–24	23	20–25
BMI				
≤ 25	22	16–24	23	19–25
26–30	22	17–24	24	21–25
≥ 31	22	16–24	23	20–25
**Surgeries**				
Isolated CABG	23	18–24	24	21–25
Single non-CABG				
Aortic valve	22	18–24	23	19–25
Mitral valve	22	17–24	23	20–25
Pulmonic/tricuspid	20	4–23	21	13–24
Aneurysectomy/aortic	16	4–21	21	14–24
Combined Surgeries				
CABG/valve ± other	19	10–23	21	15–24
Double valve	19	11–22	20	13–23
≥ 3 procedures^a^	16	2–21	19	9–22
Other^b^	21	15–24	23	17–24

^a^ ≥ 3 procedures includes triple valve, quadruple valve, double valve with aortic, CABG or aneurysectomy surgery

^b^ Other includes few combined surgeries of aortic and aneurysectomy, valve and aortic or aneuyrsectomy, CABG and aortic or aneurysectomy surgery

*CABG coronary artery bypass grafting, CCI Charlson comorbidity index, COPD chronic obstructive pulmonary disease, DAH days alive and out of hospital*

**Fig. 1:**
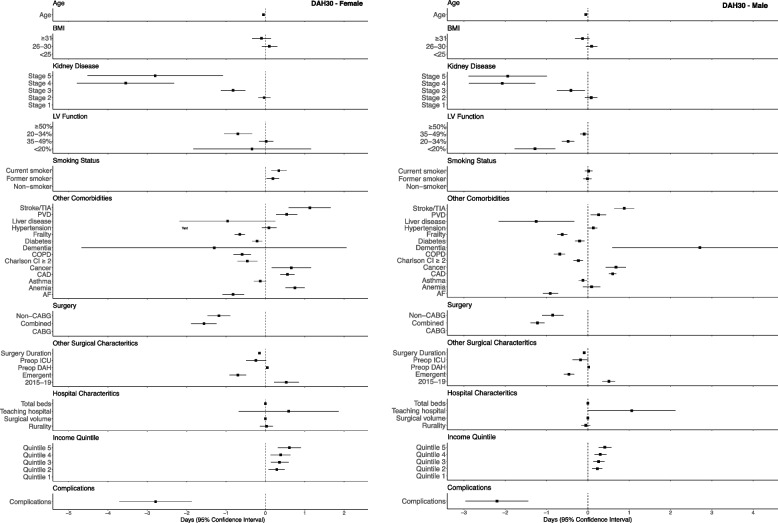
Forest plot showing risk adjusted effects of patient, surgical and hospital factors on days at alive and out of hospital (DAH) at 30 days

Risk adjusted multivariable models were conducted on 87,826 patients, 22,573 (26%) women and 65,253 (74%) men, after removal of those with missing data. Patient characteristics such as age, chronic health problems (e.g., atrial fibrillation, diabetes, chronic kidney disease, cerebro- and peripheral vascular disease, frailty), lower income quintile were all associated with reduction in DAH_30_ (Fig. 1)_._ However, there was a greater magnitude in reduction for most covariates among female patients compared to males. Complexity of surgery showed appropriate changes in DAH_30_ with greatest reduction in combined procedures followed by isolated non-CABG surgery compared to CABG. However, within each surgical group, females fared worse than males. Incurring a postoperative complication showed significant reductions in DAH_30_ by 2.8 days (95% CI -3.8 to -1.9) in females and 2.2 days in males (95%CI -3.0 to -1.4). Acuity and duration of surgery was important with urgent/emergency procedures and longer surgeries showing lower DAH_30_. Hospital factors such as size and teaching status were not associated with DAH_30_. Similar trends were seen at DAH_90_ and DAH_180_ (Supplemental Fig. S2). Sensitivity analyses after removal of patients experiencing a peri-operative complications showed similar findings (Supplemental Table S3). There was a significant interaction between patient sex and isolated non-CABG surgery (Supplemental Table S4).

After removing deaths within 30-days after surgery (*n* = 847), patients residing below the 10th percentile were undergoing more combined surgeries at an advanced age with greater comorbidity burden compared to those above the 10th percentile (Supplemental Table S5). Male and female patients below the 10th percentile showed many similar perioperative characteristics except some comorbidities (e.g., anemia, asthma, frailty) which were more common in women. Patients in the > 10th percentile group at 30-days continued to recover well with 98% and 97% remaining in this group at 90- and 180-days respectively for both female and male patients (Table 3). The proportion of patients in the < 10th group at 30-days who remained in this group at 90- and 180-days were respectively 80% and 72% for females, and 80% and 76% for males.

**Table 3:** Criterion validity of female and male patients stratified at the 10th percentile for days alive and out of hospital at 30-days assessed at 90 and 180 days

**Female patients**		**DAH**_**30**_		
		** > 10th percentile**	** ≤ 10th percentile**	**Total**
** DAH**_**90**_	** > 10th percentile**	19,630 (97.6%)	429 (19.9%)	20,059 (90.1%)
	** ≤ 10th percentile**	480 (2.4%)	1,731 (80.1%)	2,211 (9.9%)
	**Total**	20,110 (90.3%)	2,160 (9.7%)	22,270 (100)
** DAH**_**180**_	** > 10th percentile**	19,471 (96.8%)	586 (27.1%)	20,057 (90.1%)
	** ≤ 10th percentile**	639 (3.2%)	1,574 (72.9%)	2,213 (9.9%)
	**Total**	20,110 (90.3%)	2,106 (9.7%)	22,270 (100%)
**Male patients**		**DAH**_**30**_		
		** > 10th percentile**	** ≤ 10th percentile**	**Total**
** DAH**_**90**_	** > 10th percentile**	57,385 (98.3%)	1,234 (19.6%)	58,619 (90.6%)
	** ≤ 10th percentile**	1,017 (1.7%)	5,073 (80.4%)	6,090 (9.4%)
	**Total**	58,402 (90.3%)	6,307 (9.8%)	64,709 (100)
** DAH**_**180**_	** > 10th percentile**	56,783 (97.2%)	1,508 (23.9%)	58,291 (90.1%)
	** ≤ 10th percentile**	1,619 (2.8%)	4,799 (76.1%)	6,418 (9.9%)
	**Total**	58,402 (90.3%)	6,307 (9.8%)	64,709 (100%)

Among patients in the isolated non-CABG surgery, most patients underwent single aortic valve surgery, followed by mitral valve, tricuspid/pulmonic valve and aneurysectomy or aortic surgery with median DAH_30_ varying between 18–22 days for females and 22–23 days for males. Within combined procedures, most patient underwent CABG and valve surgery, multi-valve surgery or valve with aortic surgery with DAH_30_ varying 16–22 days for females and 19–23 days for males (Supplemental Table S6). In the isolated non-CABG surgery group, there was no significant difference between procedures on DAH_30_ after risk adjustment for both males and females (Supplemental Table S7). In the combined procedure group, females undergoing triple procedures showed the greatest reduction in DAH_30_ (-1.4, 95% CI -2.0 to -0.8 days) with similar but smaller finding in male patients (-0.8, 95% CI -1.3 to -0.3).

## Discussion

This observational study shows DAH is a useful instrument for evaluating the impact of surgery with appropriate and responsive change to patient comorbidities, social and economic status, surgical complexity and new complications that impact patient recovery. Similar to validation studies in noncardiac surgery, DAH is unaffected by hospital factors such as bed number and teaching status [11]. This study also shows that early outcomes at 30-days are indicative of longer-term recovery.

This study highlights sex-based disparities in patient outcomes and the importance of separate reporting of male and female adult patients in cardiac surgery. Early studies revealed differences between male and female patient outcomes in cardiovascular disease [1–3]. This study quantifies this difference and shows that patient and surgical factors impact men and women differently with the latter group showing worse outcomes. The etiology of these sex-differences requires deeper examination and is likely multifactorial with differences in technical complexity, biophysiological response to perioperative stress, and patient aging [1, 2]. There may also be sex-differences in social support and caregiver networks that facilitate hospital discharge. If women take on greater care and household duties, some women may have limited access to additional social supports that can slow hospital discharge or even lead to development of new problems within their own home. Impact of physician sex on surgical outcomes was not the aim of this study but observational studies have shown difference in patient and physician sex can negatively impact outcomes particularly among female patients and male practitioners [35, 36]. Sociocultural differences between physicians and patients may impact outcomes through sex differences in communication style and decision-making but needs further evaluation using psychosocial based research methods.

DAH provides a single metric that captures important events during the recovery pathway and articulated in simple terms of days that can be calculated using common endpoints available in healthcare datasets. The difference in DAH between men and women may appear small (i.e., 1 day at DAH_30_) but given our cohort of approximately 111,000 patients, the additional day spent in hospital by female patients (approximately 2800 days per year) has important effects on healthcare resources and bed use. DAH can be used to support patient, caregiver and physician discussions and decision making around impacts of surgery and alternative options such as less invasive percutaneous revascularization and valve procedures, medical therapy and consider combined approaches. Cardiac teams may use DAH to identify higher risk patients and those with fewer social supports to commence early discharge planning and setup of supports and services needed to aid safe hospital discharge after surgical care has been completed. DAH is a newer outcome recommended for use in perioperative care [15]. Clinicians and investigators can use DAH to study the effect of introducing new therapies, and of longitudinal and cross-sectional examination of patient outcomes between hospitals and within institutions as part of quality initiative programs. Healthcare leaders and policy makers can use DAH to examine the population-level effect of new quality care initiatives to improve healthcare processes, health policies and resource planning.

The strengths of this study include a dataset of patients over 10-years in the province of Ontario where cardiac surgical care is regionalized to 11 hospitals. This study has several limitations. This includes residual confounding from e.g., absence of physiological variables in health administrative data that may identify sicker patients. However, other variables (e.g., acuity, need for preoperative ICU level care) may provide similar information. In addition, the datasets do not capture local hospital protocols and policies for managing cardiac surgical patients. However, many aspects of cardiac care that impact patient outcomes are consistent across Canadian centres such as blood conservation techniques (e.g., use anti-fibrinolytics, cell salvage), antibiotic prophylaxis, routine advanced monitoring, ICU care after surgery, and senior physician led care. Administrative data may underestimate the true incidence and severity of complications after surgery, which may have even larger effects on DAH for men and women.

In conclusion, this study shows DAH is a useful measurement tool showing appropriate change to patient and surgical factors. There are marked sex-differences in patient outcomes and DAH should be reported separately. Further research would be valuable to understand why sex-disparities exist and what new structures and care pathways are needed to narrow this gap.

### Supplementary Information

**Additional file 1:** **Figure S1.** Calculating days alive and out of hospital at 30-days (DAH30) **Figure S2.** Forest plot showing risk adjusted effects of patient, surgical and hospital factors on days at alive and out of hospital (DAH) at (A) 90 days and (B) 180 days for female and male patients. **Table S1.** Cardiac surgery procedure codes. **Table S2.** Descriptive characteristics of cardiac surgery patients stratified by sex and surgical group. **Table S3.** Risk adjusted analysis of days alive and out of hospital for 30-, 90- and 180-days after removal of complications. **Table S4.** Summary of analyses with interaction variable. **Table S5.** Characteristics of male and female patients above and below the 10th percentile. **Table S6.** Days alive and out of hospital at 30 and 90 days for subtypes of isolated non-CABG and combined surgery stratified by patient sex. **Table S7.** Risk adjusted effects of patient, surgical and hospital factors on days at alive and out of hospital (DAH) at 30-days for women and men undergoing A) single non-CABG and B) combined surgical procedures.

## Data Availability

The data used in this manuscript is available at ICES, Toronto, ON, Canada. Enquiries to access and secondary use of the data should be made to the corresponding author and ICES and governed by ICES. The use of data in this project was authorized under section 45 of Ontario’s Personal Health Information Protection Act, which does not require review by a research ethics board.
