# Correction: Days alive and out of hospital for adult female and male cardiac surgery patients: a population-based cohort study

**DOI:** 10.1186/s12872-024-03944-6

**Published:** 2024-05-24

**Authors:** Angela Jerath, Christopher J. D. Wallis, Stephen Fremes, Vivek Rao, Terrence M. Yau, Kiyan Heybati, Douglas S. Lee, Harindra C. Wijeysundera, Jason Sutherland, Peter C. Austin, Duminda N. Wijeysundera, Dennis T. Ko

**Affiliations:** 1 Department of Anesthesia, Sunnybrook Health Sciences Center, Toronto, ON Canada; 2 Department of Anesthesiology and Pain Medicine, University of Toronto, Toronto, ON Canada; 3 ICES, 2075 Bayview Avenue, Toronto, ON Canada; 4 Schulich Heart Centre, Sunnybrook Research Institute, Sunnybrook Health Sciences Center, Toronto, ON Canada; 5 Division of Urology, Department of Surgery, University of Toronto, Toronto, ON Canada; 6 Division of Urology, Department of Surgery, Mount Sinai Hospital, Toronto, ON Canada; 7 Department of Surgical Oncology, University Health Network, Toronto, ON Canada; 8 Division of Cardiovascular Surgery, Sunnybrook Health Sciences Center, Toronto, ON Canada; 9 Division of Cardiovascular Surgery, Toronto General Hospital-University Health Network, Toronto, ON Canada; 10 Toronto General Hospital Research Institute, Toronto, ON Canada; 11 Division of Cardiovascular Surgery, University of Toronto, Toronto, ON Canada; 12 Mayo Clinic Alix School of Medicine, Mayo Clinic, Rochester, MN USA; 13 Division of Cardiology, Toronto General Hospital-University Health Network, Toronto, ON Canada; 14 Centre for Health Services and Policy Research, University of British Columbia, Vancouver, BC Canada; 15 Department of Anesthesia, St. Michael’s Hospital, Toronto, ON Canada

**Correction: BMC Cardiovascular Disorders (2024) 24:215**

10.1186/s12872-024-03862-7.

Following the publication of the original article [1], the authors notified the Acknowledgements section is not complete. Some of the vital funding acknowledgements are missing.

It was partially published as “Acknowledgements: Lu Han at ICES who assisted developing the study cohort”. The correct acknowledgement section is given below.
