# Supplementary material for: Days alive and out of hospital for adult female and male cardiac surgery patients: a population-based cohort study
Source: BMC Cardiovasc Disord. 2024 Apr 20;24:215. doi: 10.1186/s12872-024-03862-7 (PMC11031900; doi:10.1186/s12872-024-03862-7)
Supplement: Supplementary file 1 — Additional file 1: Figure S1. Calculating days alive and out of hospital at 30-days (DAH30) Figure S2. Forest plot showing risk adjusted effects of patient, surgical and hospital factors on days at alive and out of hospital (DAH) at (A) 90 days and (B) 180 days for female and male patients. Table S1. Cardiac surgery procedure codes. Table S2. Descriptive characteristics of cardiac surgery patients stratified by sex and surgical group. Table S3. Risk adjusted analysis of days alive and out of hospital for 30-, 90- and 180-days after removal of complications. Table S4. Summary of analyses with interaction variable. Table S5. Characteristics of male and female patients above and below the 10th percentile. Table S6. Days alive and out of hospital at 30 and 90 days for subtypes of isolated non-CABG and combined surgery stratified by patient sex. Table S7. Risk adjusted effects of patient, surgical and hospital factors on days at alive and out of hospital (DAH) at 30-days for women and men undergoing A) single non-CABG and B) combined surgical procedures. [file 12872_2024_3862_MOESM1_ESM.docx]

**SUPPLEMENTAL FILE**

**Title: Days Alive and Out of Hospital for Adult Female and Male Cardiac Surgery patients: A population-based cohort study.**

**Authors:** Angela Jerath MD, MSc, FRCPC, FANZCA^1-4^, Christopher JD Wallis MD PhD FRCSC^5-7^, Stephen Fremes MD FRCSC^4,8,11^, Vivek Rao MD PhD FRCSC^9,10,11^, Terrence M. Yau MD MSc FRCSC^10,11^, Kiyan Heybati BHSc^12^, Douglas S Lee MD PhD FRCPC^3,10,13^, Harindra C Wijeysundera MD PhD FRCPC^3,4^, Jason Sutherland PhD^14^, Peter C Austin PhD^3^, Duminda N Wijeysundera MD PhD FRCPC^3,15^, Dennis T Ko. MD, MSc FRCPC^3,4^

**Address:**

^1^Department of Anesthesia, Sunnybrook Health Sciences Center, Toronto, ON, Canada

^2^Department of Anesthesiology and Pain Medicine, University of Toronto, Toronto, ON, Canada

^3^ ICES, 2075 Bayview Avenue, Toronto, ON, Canada

^4^Schulich Heart Centre, Sunnybrook Research Institute, Sunnybrook Health Sciences Center, Toronto, ON, Canada

^5^Division of Urology, Department of Surgery, University of Toronto, Toronto, ON, Canada

^6^Division of Urology, Department of Surgery, Mount Sinai Hospital, Toronto, ON, Canada

^7^Department of Surgical Oncology, University Health Network, Toronto, ON, Canada

^8^Division of Cardiovascular Surgery, Sunnybrook Health Sciences Center, Toronto, ON, Canada

^9^Division of Cardiovascular Surgery, Toronto General Hospital-University Health Network, Toronto, ON, Canada

^10^Toronto General Hospital Research Institute, Toronto, ON, Canada

^11^Division of Cardiovascular Surgery, University of Toronto, Toronto, ON, Canada

^12^Mayo Clinic Alix School of Medicine, Mayo Clinic, Rochester, MN, USA

^13^Division of Cardiology, Toronto General Hospital-University Health Network, Toronto, ON, Canada

^14^Centre for Health Services and Policy Research, University of British Columbia, Vancouver, BC, Canada

^15^Department of Anesthesia, St. Michael’s Hospital, Toronto, ON, Canada

**Figure 1.** Calculating days alive and out of hospital at 30-days (DAH_30_).

**Figure 2.** Forest plot showing risk adjusted effects of patient, surgical and hospital factors on days at alive and out of hospital (DAH) at (A) 90 days and (B) 180 days for female and male patients.

**Table 1.** Cardiac surgery procedure codes.

**Table 2.** Descriptive characteristics of cardiac surgery patients stratified by sex and surgical group.

**Table 3.** Risk adjusted analysis of days alive and out of hospital for 30-, 90- and 180-days after removal of complications.

**Table 4.** Summary of analyses with interaction variable.

**Table 5.** Characteristics of male and female patients above and below the 10^th^ percentile.

**Table 6.** Days alive and out of hospital at 30 and 90 days for subtypes of isolated non-CABG and combined surgery stratified by patient sex.

**Table 7.** Risk adjusted effects of patient, surgical and hospital factors on days at alive and out of hospital (DAH) at 30-days for women and men undergoing A) single non-CABG and B) combined surgical procedures.

**Figure 1. Calculating days alive and out of hospital at 30-days (DAH_30_)**

**
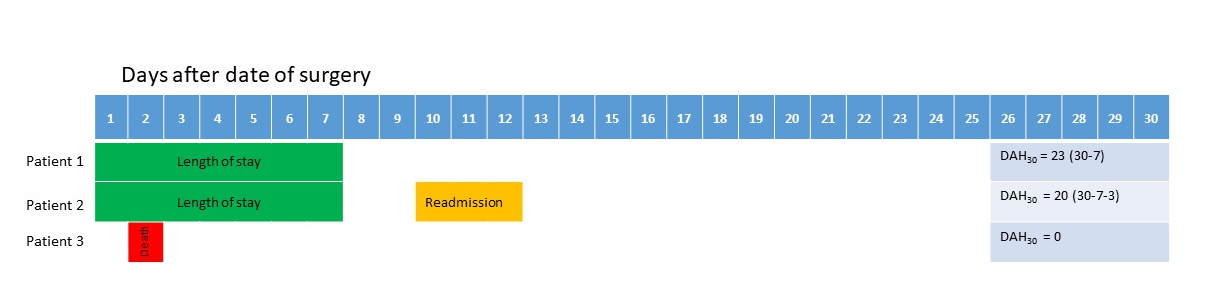
**

**Figure 2. Forest plot showing risk adjusted effects of patient, surgical and hospital factors on days at alive and out of hospital (DAH) at (A) 90 days and (B) 180 days for female and male patients.**

**A. DAH_90_**

**
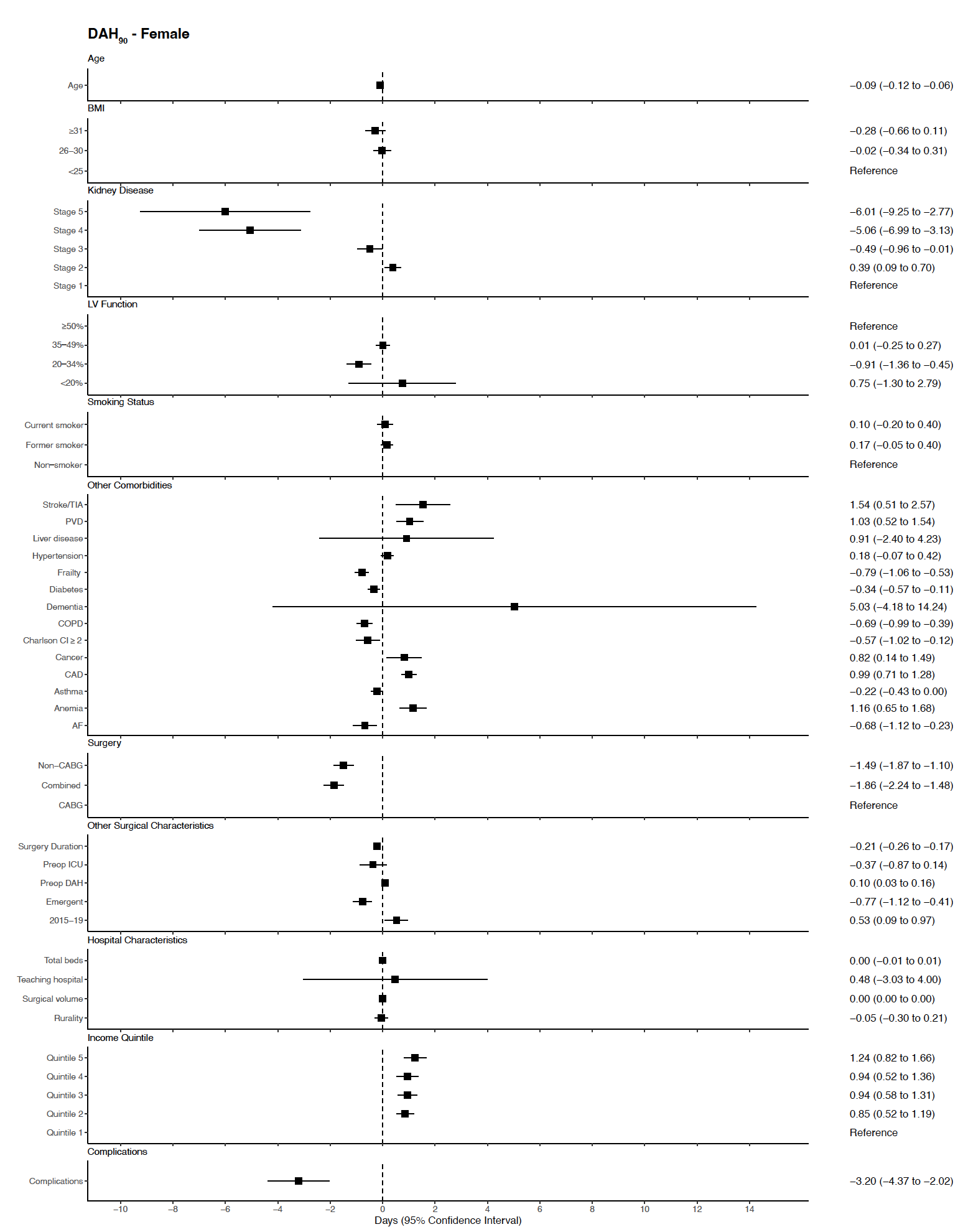
**

**
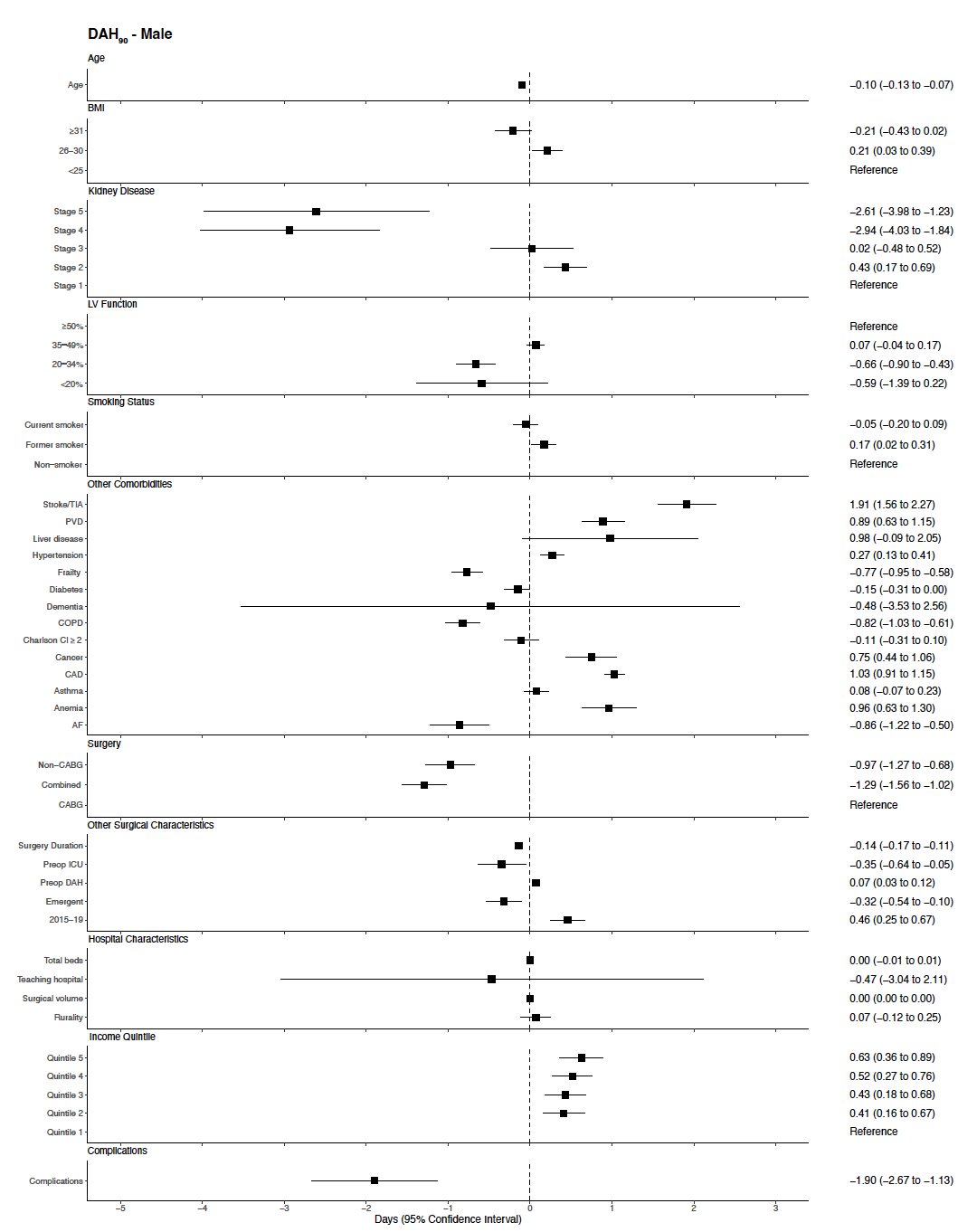
**

**B.** **DAH_180_**

**
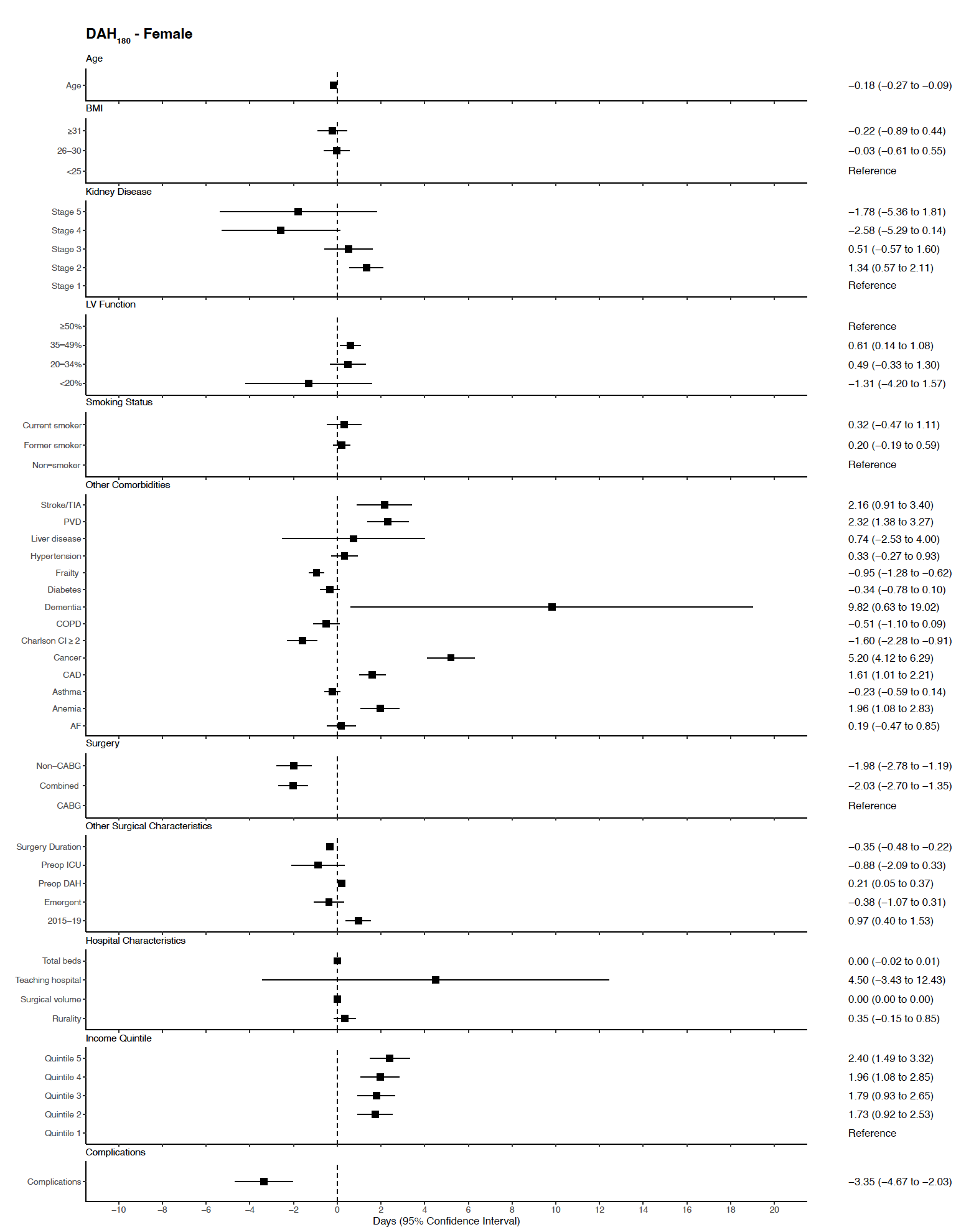
**

**
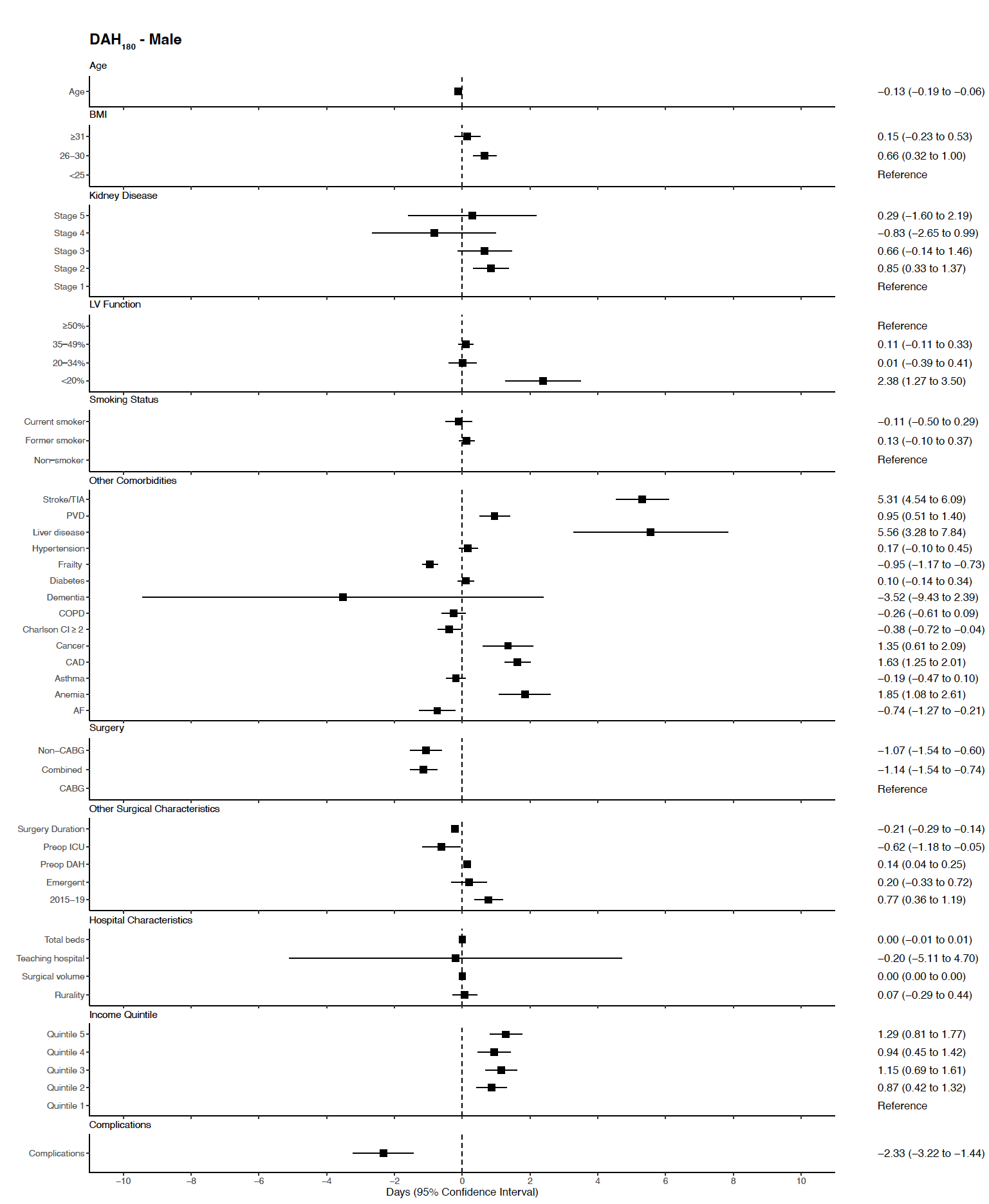
**

**Table 1. Cardiac surgery procedure codes.**

|  | **Definition / Database** | **Code / other** |
| --- | --- | --- |
| **SURGICAL INTERVENTIONS** | | |
| CABG only | CorHealth  DAD | Isolated CABG (on-pump or off-pump) surgery only:  BYPASS SURGERY (420)  (should not include Aortic Valve Surgery (422), Mitral Valve Surgery (424), Tricuspid Valve Surgery (426), Pulmonary Valve Surgery (428), Aortic Surgery (435), Aneurysmectomy (437)  Any of following: 1.IJ.76 (all codes), 1.IJ.80, 1.IJ.86 |
| Single non-CABG surgery | CorHealth  DAD | Isolated single valve surgery. Only 1 of the following codes: Aortic Valve Surgery (422), Mitral Valve Surgery (424), Tricuspid Valve Surgery (426), Pulmonary Valve Surgery (428)  Only 1 of the following valves:  TV: 1.HS.80 (all codes), 1.HS.90 (all codes)  PV: 1.HT.80 (all codes), 1.HT.89, 1.HT.90  MV: 1.HU.80 (all codes), 1.HU.90 (all codes),  AV: 1.HV.80 (all codes), 1.HV.90 |
| Combined procedures | CorHealth  DAD | 2 or more procedures from CABG, valve, aorta, LV aneurysm or multiple (2 or more) valves.  BYPASS SURGERY (420), Aortic Valve Surgery (422), Mitral Valve Surgery (424), Tricuspid Valve Surgery (426), Pulmonary Valve Surgery (428), Aortic Surgery (435), Aneurysmectomy (437)  CABG: 1.IJ.76 (all codes), 1.IJ.80, 1.IJ.86  VALVE: 1.HS.80 (all codes), 1.HS.90 (all codes), 1.HT.80 (all codes), 1.HT.89, 1.HT.90, 1.HU.80 (all codes), 1.HU.90 (all codes), 1.HV.80 (all codes), 1.HV.90, 1.HW.78, 1.HW.79, 1.HX.71, 1.HX.78, 1.HX.79, 1. HX.80, 1.HX.83, 1.HX.86, 1.HX.87  AORTIC: 1.IA.57, , 1.IA.79, 1.IA.80, 1.IA.86, 1.IA.87, 1.IB.57, 1.IB.76, 1.IB.80, 1.IB.82, 1.IB.87,  VAD: 1.HP.53 1  Ventricle procedures: 1.HP.78, 1.HP.87 |

**Table 2. Descriptive characteristics of cardiac surgery patients stratified by sex and surgical group.**

|  | **Isolated CABG** | | **Isolated non-CABG** | | **Combined surgeries** | | **Total** | |
| --- | --- | --- | --- | --- | --- | --- | --- | --- |
|  | **Female** | **Male** | **Female** | **Male** | **Female** | **Male** | **Female** | **Male** |
| **Patient factors** | **N=14,111** | **N=56,343** | **N=6,933** | **N=10,298** | **N=7,393** | **N=16,352** | **N=28,437** | **N=82,993** |
| Age | 69 (61-75) | 66 (59-73) | 69 (59-76) | 65 (55-74) | 72 (63-78) | 70 (61-77) | 69 (61-76) | 66 (59-74) |
| Atrial fibrillation | 979 (6.9%) | 4,072 (7.2%) | 1,388 (20.0%) | 1,816 (17.6%) | 1,790 (24.2%) | 3,046 (18.6%) | 4,157 (14.6%) | 8,934 (10.8%) |
| Anemia | 1,343 (9.5%) | 2,625 (4.7%) | 744 (10.7%) | 747 (7.3%) | 912 (12.3%) | 1,434 (8.8%) | 2,999 (10.5%) | 4,806 (5.8%) |
| Asthma | 2,523 (17.9%) | 5,578 (9.9%) | 1,408 (20.3%) | 1,327 (12.9%) | 1,392 (18.8%) | 1,865 (11.4%) | 5,323 (18.7%) | 8,770 (10.6%) |
| CAD | 9,489 (67.2%) | 33,918 (60.2%) | 815 (11.8%) | 1,410 (13.7%) | 4,047 (54.7%) | 10,733 (65.6%) | 14,351 (50.5%) | 46,061 (55.5%) |
| Stroke | 368 (2.6%) | 1,251 (2.2%) | 212 (3.1%) | 293 (2.8%) | 226 (3.1%) | 546 (3.3%) | 806 (2.8%) | 2,090 (2.5%) |
| Dementia | 42 (0.3%) | 100 (0.2%) | 14 (0.2%) | 22 (0.2%) | 27 (0.4%) | 59 (0.4%) | 83 (0.3%) | 181 (0.2%) |
| Diabetes | 7,660 (54.3%) | 26,011 (46.2%) | 2,040 (29.4%) | 2,916 (28.3%) | 2,691 (36.4%) | 5,954 (36.4%) | 12,391 (43.6%) | 34,881 (42.0%) |
| Dialysis | 266 (1.9%) | 872 (1.5%) | 76 (1.1%) | 161 (1.6%) | 133 (1.8%) | 266 (1.6%) | 475 (1.7%) | 1,299 (1.6%) |
| Hypertension | 12,808 (90.8%) | 47,851 (84.9%) | 5,023 (72.5%) | 7,228 (70.2%) | 6,130 (82.9%) | 13,379 (81.8%) | 23,961 (84.3%) | 68,458 (82.5%) |
| Chronic liver disease | 55 (0.4%) | 265 (0.5%) | 90 (1.3%) | 166 (1.6%) | 71 (1.0%) | 186 (1.1%) | 216 (0.8%) | 617 (0.7%) |
| Myocardial infarction | 5,885 (41.7%) | 20,233 (35.9%) | 274 (4.0%) | 494 (4.8%) | 988 (13.4%) | 2,599 (15.9%) | 7,147 (25.1%) | 23,326 (28.1%) |
| PVD | 472 (3.3%) | 1,583 (2.8%) | 246 (3.5%) | 470 (4.6%) | 1,140 (15.4%) | 3,200 (19.6%) | 1,858 (6.5%) | 5,253 (6.3%) |
| Primary cancer | 295 (2.1%) | 1,298 (2.3%) | 172 (2.5%) | 303 (2.9%) | 194 (2.6%) | 517 (3.2%) | 661 (2.3%) | 2,118 (2.6%) |
| Secondary cancer | 42 (0.3%) | 155 (0.3%) | 34 (0.5%) | 36 (0.3%) | 33 (0.4%) | 55 (0.3%) | 109 (0.4%) | 246 (0.3%) |
| COPD | 1,684 (11.9%) | 5,106 (9.1%) | 762 (11.0%) | 1,050 (10.2%) | 1,010 (13.7%) | 2,012 (12.3%) | 3,456 (12.2%) | 8,168 (9.8%) |
| Chronic kidney disease |  |  |  |  |  |  |  |  |
| Stage 1 | 3,435 (24.3%) | 16,925 (30.0%) | 1,635 (23.6%) | 3,020 (29.3%) | 1,079 (14.6%) | 3,002 (18.4%) | 6,149 (21.6%) | 22,947 (27.6%) |
| Stage 2 | 5,286 (37.5%) | 21,764 (38.6%) | 2,748 (39.6%) | 3,987 (38.7%) | 2,913 (39.4%) | 6,668 (40.8%) | 10,947 (38.5%) | 32,419 (39.1%) |
| Stage 3 | 2,744 (19.4%) | 7,808 (13.9%) | 1,447 (20.9%) | 1,608 (15.6%) | 2,058 (27.8%) | 3,870 (23.7%) | 6,249 (22.0%) | 13,286 (16.0%) |
| Stage 4 | 452 (3.2%) | 1,088 (1.9%) | 202 (2.9%) | 269 (2.6%) | 381 (5.2%) | 634 (3.9%) | 1,035 (3.6%) | 1,991 (2.4%) |
| Stage 5 | 225 (1.6%) | 646 (1.1%) | 44 (0.6%) | 90 (0.9%) | 75 (1.0%) | 167 (1.0%) | 344 (1.2%) | 903 (1.1%) |
| CCI ≥ 2 | 5,592 (39.6%) | 17,086 (30.3%) | 1,264 (18.2%) | 1,873 (18.2%) | 2,171 (29.4%) | 4,816 (29.5%) | 9,027 (31.7%) | 23,775 (28.6%) |
| Rural | 2,104 (14.9%) | 8,272 (14.7%) | 1,031 (14.9%) | 1,664 (16.2%) | 1,102 (14.9%) | 2,603 (15.9%) | 4,237 (14.9%) | 12,539 (15.1%) |
| Income quintile |  |  |  |  |  |  |  |  |
| Q1 | 3,364 (23.9%) | 8,112 (14.4%) | 1,414 (20.4%) | 1,711 (16.7%) | 1,506 (20.4%) | 2,732 (16.8%) | 6,284 (22.2%) | 14,752 (17.8%) |
| Q2 | 3,044 (21.7%) | 16,925 (30.0%) | 1,416 (20.5%) | 1,983 (19.3%) | 1,592 (21.6%) | 3,175 (19.5%) | 6,052 (21.4%) | 16,582 (20.0%) |
| Q3 | 2,855 (20.3%) | 21,764 (38.6%) | 1,358 (19.6%) | 2,072 (20.2%) | 1,464 (19.9%) | 3,340 (20.5%) | 5,677 (20.0%) | 17,057 (20.6%) |
| Q4 | 2,467 (17.6%) | 7,808 (13.9%) | 1,343 (19.4%) | 2,128 (20.7%) | 1,458 (19.8%) | 3,450 (21.2%) | 5,268 (18.6%) | 17,156 (20.7%) |
| Q5 | 2,317 (16.5%) | 1,088 (1.9%) | 1,385 (20.0%) | 2,370 (23.1%) | 1,353 (18.4%) | 3,608 (22.1%) | 5,055 (17.8%) | 17,162 (20.7%) |
| Body mass index |  |  |  |  |  |  |  |  |
| ≤ 25 | 3,509 (24.9%) | 646 (1.1%) | 2,259 (32.6%) | 2,664 (25.9%) | 2,438 (33.0%) | 4,042 (24.7%) | 8,206 (28.9%) | 19,187 (23.1%) |
| 26-30 | 4,501 (31.9%) | 17,086 (30.3%) | 1,991 (28.7%) | 3,934 (38.2%) | 2,082 (28.2%) | 6,388 (39.1%) | 8,574 (30.2%) | 32,880 (39.6%) |
| ≥ 31 | 5,398 (38.3%) | 8,272 (14.7%) | 2,314 (33.4%) | 3,139 (30.5%) | 2,562 (34.7%) | 5,200 (31.8%) | 10,274 (36.1%) | 26,736 (32.2%) |
| LVEF |  |  |  |  |  |  |  |  |
| <20% | 160 (1.1%) | 1,061 (1.9%) | 30 (0.4%) | 107 (1.0%) | 51 (0.7%) | 341 (2.1%) | 241 (0.8%) | 1,509 (1.8%) |
| 20% - 34% | 1,149 (8.1%) | 5,337 (9.5%) | 172 (2.5%) | 484 (4.7%) | 394 (5.3%) | 1,369 (8.4%) | 1,715 (6.0%) | 7,190 (8.7%) |
| 35% - 49% | 2,854 (20.2%) | 13,672 (24.3%) | 531 (7.7%) | 1,174 (11.4%) | 993 (13.4%) | 2,703 (16.5%) | 4,378 (15.4%) | 17,549 (21.1%) |
| ≥ 50% | 9,357 (66.3%) | 34,245 (60.8%) | 6,067 (87.5%) | 8,289 (80.5%) | 5,809 (78.6%) | 11,535 (70.5%) | 21,233 (74.7%) | 54,069 (65.1%) |
| NYHA |  |  |  |  |  |  |  |  |
| 1 | 5,072 (35.9%) | 20,775 (36.9%) | 1,585 (22.9%) | 3,091 (30.0%) | 1,593 (21.5%) | 4,665 (28.5%) | 8,250 (29.0%) | 28,531 (34.4%) |
| 2 | 1,221 (8.7%) | 4,717 (8.4%) | 2,101 (30.3%) | 3,137 (30.5%) | 1,819 (24.6%) | 4,465 (27.3%) | 5,141 (18.1%) | 12,319 (14.8%) |
| 3 | 869 (6.2%) | 2,710 (4.8%) | 2,255 (32.5%) | 2,547 (24.7%) | 2,584 (35.0%) | 4,059 (24.8%) | 5,708 (20.1%) | 9,316 (11.2%) |
| 4 | 274 (1.9%) | 725 (1.3%) | 428 (6.2%) | 560 (5.4%) | 668 (9.0%) | 1,093 (6.7%) | 1,370 (4.8%) | 2,378 (2.9%) |
| Unknown | 6,675 (47.3%) | 27,416 (48.7%) | 564 (8.1%) | 963 (9.4%) | 729 (9.9%) | 2,070 (12.7%) | 7,968 (28.0%) | 30,449 (36.7%) |
| Smoking status |  |  |  |  |  |  |  |  |
| Current | 2,784 (19.9%) | 12,488 (22.4%) | 834 (12.2%) | 1,556 (15.3%) | 905 (12.3%) | 2,638 (16.3%) | 4,523 (16.1%) | 16,682 (20.3%) |
| Former | 3,557 (25.4%) | 21,206 (38.0%) | 1,464 (21.4%) | 3,392 (33.3%) | 1,778 (24.3%) | 6,633 (40.9%) | 6,799 (24.1%) | 31,231 (38.0%) |
| Never | 7,480 (53.5%) | 21,581 (38.7%) | 4,477 (65.3%) | 5,091 (50.0%) | 4,597 (62.7%) | 6,800 (41.9%) | 16,554 (58.8%) | 33,472 (40.7%) |
| Unknown | 157 (1.1%) | 549 (1.0%) | 78 (1.1%) | 133 (1.3%) | 51 (0.7%) | 144 (0.9%) | 286 (1.0%) | 826 (1.0%) |
| Frailty | 2 (0-5) | 1 (0-3) | 1 (0-4) | 0 (0-3) | 2 (0-6) | 2 (0-5) | 2 (0-5) | 1 (0-3) |
| Preop DAH (3m - 1d) | 86 (81-91) | 88 (83-92) | 91 (87-92) | 91 (87-92) | 90 (83-92) | 90 (83-92) | 89 (82-92) | 89 (83-92) |
| **Surgery** |  |  |  |  |  |  |  |  |
| Surgery duration (min) | 258 (220-305) | 264 (225-310) | 252 (210-305) | 265 (218-321) | 308 (254-378) | 323 (267-391) | 268 (223-323) | 273 (230-326) |
| Preop ICU level care | 8,264 (58.6%) | 33,151 (58.8%) | 3,686 (53.2%) | 5,358 (52.0%) | 3,963 (53.6%) | 8,849 (54.1%) | 15,913 (56.0%) | 47,358 (57.1%) |
| Elective | 7,039 (49.9%) | 31,333 (55.6%) | 5,621 (81.1%) | 8,402 (81.6%) | 5,315 (71.9%) | 11,581 (70.8%) | 17,975 (63.2%) | 51,316 (61.8%) |
| Urgent/Emergent | 7,072 (50.1%) | 25,010 (44.4%) | 1,312 (18.9%) | 1,896 (18.4%) | 2,078 (28.1%) | 4,771 (29.2%) | 10,462 (36.8%) | 31,677 (38.2%) |
| **Hospital factors** |  |  |  |  |  |  |  |  |
| Teaching hospital | 8,564 (60.7%) | 33,707 (59.8%) | 4,591 (66.2%) | 7,013 (68.1%) | 4,479 (60.6%) | 9,811 (60.0%) | 17,634 (62.0%) | 50,531 (60.9%) |
| ICU Beds | 60 (35-70) | 60 (35-70) | 64 (35-79) | 64 (35-85) | 61 (35-77) | 61 (35-77) | 61 (35-77) | 60 (35-74) |
| Surgical Beds | 131 (85-171) | 131 (85-171) | 134 (84-229) | 134 (85-238) | 131 (72-248) | 131 (77-221) | 131 (84-210) | 131 (84-176) |
| Total Beds | 367 (291-507) | 373 (291-507) | 416 (291-554) | 403 (291-717) | 360 (274-722) | 360 (277-536) | 367 (288-514) | 367 (291-513) |
| **Outcomes** |  |  |  |  |  |  |  |  |
| 30-day mortality | 129 (0.9%) | 310 (0.6%) | 93 (1.3%) | 95 (0.9%) | 196 (2.7%) | 373 (2.3%) | 418 (1.5%) | 778 (0.9%) |
| 90-day mortality | 214 (1.5%) | 527 (0.9%) | 147 (2.1%) | 158 (1.5%) | 309 (4.2%) | 553 (3.4%) | 670 (2.4%) | 1,238 (1.5%) |
| ICU LOS (h) | 44 (24-85) | 30 (23-70) | 42 (24-80) | 29 (23-71) | 63 (27-123) | 50 (25-114) | 47 (25-95) | 37 (23-75) |
| ALC patient | 595 (4.2%) | 1,214 (2.2%) | 358 (5.2%) | 293 (2.8%) | 427 (5.8%) | 648 (4.0%) | 1,380 (4.9%) | 2,155 (2.6%) |
| Postop LOS (d) | 7 (5-9) | 6 (5-7) | 7 (6-10) | 6 (5-9) | 9 (6-14) | 8 (6-12) | 7 (6-10) | 6 (5-8) |
| Major complications | 2,238 (15.9%) | 7,347 (13.0%) | 1,601 (23.1%) | 2,136 (20.7%) | 2,309 (31.2%) | 4,649 (28.4%) | 6,148 (21.6%) | 14,132 (17.0%) |
| ^a^DAH_30_ | 23 (18-24) | 24 (21-25) | 22 (17-24) | 23 (19-25) | 19 (11-23) | 21 (15-24) | 22 (16-24) | 23 (20-25) |
|  | 19.7 ± 7.1 | 21.8 ± 5.8 | 19.0 ± 7.4 | 20.4 ± 6.8 | 15.9 ± 8.6 | 17.7 ± 8.2 | 18.5 ± 7.8 | 20.8 ± 6.7 |
| ^a^DAH_90_ | 82 (76-84) | 84 (81-85) | 81 (75-84) | 83 (78-85) | 78 (67-82) | 81 (72-84) | 81 (74-84) | 83 (79-85) |
|  | 75.2 ± 18.6 | 79.3 ± 14.3 | 74.2 ± 19.3 | 77.0 ± 16.8 | 67.6 ± 24.6 | 71.5 ± 22.3 | 73.0 ± 20.7 | 77.5 ± 16.8 |
| ^a^DAH_180_ | 172 (165-174) | 173 (170-175) | 171 (164-174) | 172 (167-174) | 167 (154-172) | 170 (160-173) | 171 (162-173) | 173 (168-175) |
|  | 160.0 ± 35.4 | 166.3 ± 27.1 | 158.6 ± 36.7 | 162.9 ± 31.6 | 147.6 ± 48.3 | 153.7 ± 43.7 | 156.5 ± 39.8 | 163.4 ± 32.0 |

*ALC alternate care; CAD coronary artery disease; CCI Charlson comorbidity index; COPD chronic obstructive pulmonary disease; d days; DAH days alive and out of hospital; h hours; ICU intensive care unit; LOS length of stay; LVEF left ventricular ejection fraction; m month; NYHA New York Heart Association; PVD peripheral vascular disease*

*^a^DAH described using median (inter-quartile range) and mean ± standard deviation*

**Table 3. Risk adjusted analysis of days alive and out of hospital for 30-, 90- and 180-days after removal of complications.**

| 1. **Days alive and out of hospital at 30-days** | | | | | | | | | | | |
| --- | --- | --- | --- | --- | --- | --- | --- | --- | --- | --- | --- |
| **Female** |  |  |  |  |  |  | **Males** |  |  |  |  |
| **Variable** | **Parameter estimate (days)** | **St Err** | **95% LCL** | **95% UCL** | **P-value** |  | **Parameter estimate (days)** | **St Err** | **95% LCL** | **95% UCL** | **P-value** |
| Surgery |  |  |  |  |  |  |  |  |  |  |  |
| CABG | Ref |  |  |  |  |  |  |  |  |  |  |
| Non-CABG | -1.31 | 0.15 | -1.61 | -1.00 | <.001 |  | -0.88 | 0.13 | -1.13 | -0.61 | <.001 |
| Combined | -1.71 | 0.16 | -2.03 | -1.4 | <.001 |  | -1.38 | 0.10 | -1.58 | -1.17 | <.001 |
| Age | -0.06 | 0.01 | -0.07 | -0.04 | <.001 |  | -0.06 | 0.01 | -0.08 | -0.04 | <.001 |
| Preop DAH | 0.02 | 0.01 | 0.00 | 0.04 | 0.07 |  | -0.02 | 0.01 | -0.03 | 0.00 | 0.09 |
| Surgical volume | 0.00 | 0.00 | 0.00 | 0 | 0.56 |  | 0.00 | 0.00 | 0.00 | 0.00 | 0.34 |
| Rurality | 0.10 | 0.00 | -0.06 | 0.25 | 0.22 |  | 0.02 | 0.06 | -0.11 | 0.15 | 0.79 |
| Income Quintile |  |  |  |  |  |  |  |  |  |  |  |
| Quintile 1 | Ref |  |  |  |  |  |  |  |  |  |  |
| Quintile 2 | 0.19 | 0.11 | -0.02 | 0.41 | 0.08 |  | 0.36 | 0.07 | 0.12 | 0.41 | <.001 |
| Quintile 3 | 0.39 | 0.11 | 0.17 | 0.61 | <.001 |  | 0.34 | 0.06 | 0.22 | 0.46 | <.001 |
| Quintile 4 | 0.37 | 0.12 | 0.13 | 0.60 | <.01 |  | 0.40 | 0.05 | 0.29 | 0.51 | <.001 |
| Quintile 5 | 0.60 | 0.12 | 0.36 | 0.85 | <.001 |  | 0.50 | 0.07 | 0.36 | 0.64 | <.001 |
| Surgery Duration^a^ | -0.16 | 0.01 | -0.19 | -0.13 | <.001 |  | -0.10 | 0.01 | -0.12 | -0.08 | <.001 |
| Surgery Urgency |  |  |  |  |  |  |  |  |  |  |  |
| Elective | Ref |  |  |  |  |  |  |  |  |  |  |
| Emergent | -0.53 | 0.10 | -0.73 | -0.32 | <.001 |  | -0.38 | 0.05 | -0.49 | -0.28 | <.001 |
| Total beds | 0.00 | 0.00 | 0.00 | 0.00 | 0.91 |  | 0.00 | 0.00 | 0.00 | 0.00 | 0.59 |
| Preop ICU | -0.10 | 0.15 | -0.39 | 0.19 | 0.51 |  | -0.14 | 0.09 | -0.33 | 0.05 | 0.15 |
| Year |  |  |  |  |  |  |  |  |  |  |  |
| 2009-14 | Ref |  |  |  |  |  |  |  |  |  |  |
| 2015-19 | 0.53 | 0.25 | 0.23 | 0.83 | <.001 |  | 0.50 | 0.08 | 0.34 | 0.66 | <.001 |
| Teaching hospital | 0.45 | 0.60 | -0.73 | 1.63 | 0.45 |  | 0.30 | 0.53 | -0.75 | 1.35 | 0.57 |
| BMI |  |  |  |  |  |  |  |  |  |  |  |
| <25 | Ref |  |  |  |  |  |  |  |  |  |  |
| 26-30 | 0.11 | 0.08 | -0.06 | 0.28 | 0.20 |  | 0.07 | 0.07 | -0.06 | 0.20 | 0.30 |
| ≥ 31 | -0.12 | 0.11 | -0.33 | 0.09 | 0.27 |  | -0.15 | 0.09 | -0.33 | 0.02 | 0.09 |
| Smoking status |  |  |  |  |  |  |  |  |  |  |  |
| Non-smoker | Ref |  |  |  |  |  |  |  |  |  |  |
| Former smoker | 0.20 | 0.07 | 0.06 | 0.34 | <.01 |  | 0.05 | 0.05 | -0.05 | 0.14 | 0.30 |
| Current smoker | 0.36 | 0.09 | 0.18 | 0.55 | <.001 |  | 0.01 | 0.04 | -0.07 | 0.09 | 0.81 |
| Charlson CI ≥ 2 | -0.38 | 0.10 | -0.58 | -0.18 | <.001 |  | -0.19 | 0.06 | -0.31 | -0.09 | <.001 |
| Asthma | -0.18 | 0.07 | -0.31 | -0.03 | <.001 |  | -0.15 | 0.05 | -0.25 | -0.05 | <.01 |
| CAD | 0.55 | 0.08 | 0.40 | 0.70 | <.001 |  | 0.54 | 0.04 | 0.47 | 0.62 | <.001 |
| AF | -0.78 | 0.19 | -1.15 | -0.40 | <.001 |  | -0.92 | 0.10 | -1.13 | -0.71 | <.001 |
| PVD | 0.48 | 0.15 | 0.17 | 0.79 | <.01 |  | 0.34 | 0.09 | 0.17 | 0.52 | <.001 |
| Stroke/TIA | 1.01 | 0.34 | 0.33 | 1.69 | <.01 |  | 0.91 | 0.15 | 0.62 | 1.20 | <.001 |
| COPD | -0.68 | 0.10 | -0.87 | -0.48 | <.001 |  | -0.57 | 0.06 | -0.68 | -0.45 | <.001 |
| Hypertension | 0.20 | 0.09 | 0.02 | 0.37 | <.05 |  | 0.00 | 0.05 | -0.10 | 0.09 | 0.99 |
| Kidney disease |  |  |  |  |  |  |  |  |  |  |  |
| Stage 1 | Ref |  |  |  |  |  |  |  |  |  |  |
| Stage 2 | -0.10 | 0.07 | -0.24 | 0.05 | 0.20 |  | 0.11 | 0.07 | -0.02 | 0.25 | 0.10 |
| Stage 3 | -1.01 | 0.12 | -1.25 | -0.76 | <.001 |  | -0.33 | 0.16 | -0.64 | -0.02 | <.05 |
| Stage 4 | -3.53 | 0.56 | -4.65 | -2.42 | <.001 |  | -2.08 | 0.41 | -2.88 | -1.27 | <.001 |
| Stage 5 | -3.64 | 0.88 | -5.39 | -1.89 | <.001 |  | -2.17 | 0.47 | -3.09 | -1.24 | <.001 |
| Diabetes | -0.20 | 0.07 | -0.34 | -0.07 | <.01 |  | -0.15 | 0.06 | -0.28 | -0.03 | <.05 |
| Dementia | 0.25 | 1.78 | -3.29 | 3.79 | 0.89 |  | 2.34 | 1.06 | 0.23 | 4.44 | <.05 |
| Liver disease | -1.10 | 0.66 | -2.40 | 0.21 | 0.10 |  | -0.26 | 0.48 | -1.21 | 0.70 | 0.59 |
| Cancer | 0.37 | 0.29 | -0.20 | 0.94 | 0.20 |  | 0.63 | 0.10 | 0.22 | 0.81 | <.001 |
| LV Function |  |  |  |  |  |  |  |  |  |  |  |
| ≥ 50% | Ref |  |  |  |  |  |  |  |  |  |  |
| 35-49% | 0.02 | 0.22 | -0.21 | 0.24 | 0.89 |  | -0.13 | 0.05 | -0.22 | -0.03 | <.05 |
| 20-34% | -0.76 | 0.21 | -1.18 | -0.35 | <.001 |  | -0.44 | 0.08 | -0.60 | -0.29 | <.001 |
| <20% | -0.59 | 0.57 | -1.73 | 0.55 | 0.30 |  | -0.89 | 0.30 | -1.48 | -0.30 | <.01 |
| Anemia | 0.92 | 0.15 | 0.63 | 1.21 | <.001 |  | 0.21 | 0.12 | -3.31 | 0.45 | 0.09 |
| Frailty | -0.76 | 0.07 | -0.90 | -0.61 | <.001 |  | -0.73 | 0.07 | -0.87 | -0.59 | <.001 |

| 1. **Days alive and out of hospital at 90-days** | | | | | | | | | |  |  |
| --- | --- | --- | --- | --- | --- | --- | --- | --- | --- | --- | --- |
| **Female** |  |  |  |  |  |  | **Males** |  |  |  |  |
| **Variable** | **Parameter estimate (days)** | **St Err** | **95% LCL** | **95% UCL** | **P-value** |  | **Parameter estimate (days)** | **St Err** | **95% LCL** | **95% UCL** | **P-value** |
| Surgery |  |  |  |  |  |  |  |  |  |  |  |
| CABG | Ref |  |  |  |  |  |  |  |  |  |  |
| Non-CABG | -2.04 | 0.22 | -2.47 | -1.61 | <.001 |  | -1.23 | 0.15 | -1.53 | -0.93 | <.001 |
| Combined | -2.58 | 0.24 | -3.04 | -2.11 | <.001 |  | -1.43 | 0.15 | -1.73 | -1.14 | <.001 |
| Age | -0.10 | 0.02 | -0.14 | -0.07 | <.001 |  | -0.1 | 0.01 | -0.13 | -0.07 | <.001 |
| Preop DAH | 0.04 | 0.03 | -0.03 | 0.1 | 0.25 |  | 0.02 | 0.02 | -0.03 | 0.06 | 0.50 |
| Surgical volume | 0.00 | 0.00 | -0.03 | 0.1 | 0.25 |  | 0.00 | 0.00 | 0.00 | 0.00 | 0.08 |
| Rurality | -0.03 | 0.15 | -0.33 | 0.27 | 0.84 |  | 0.19 | 0.09 | 0.01 | 0.37 | <.05 |
| Income Quintile |  |  |  |  |  |  |  |  |  |  |  |
| Quintile 1 | Ref |  |  |  |  |  |  |  |  |  |  |
| Quintile 2 | 0.65 | 0.18 | 0.29 | 1 | <.001 |  | 0.59 | 0.13 | 0.33 | 0.84 | <.001 |
| Quintile 3 | 0.83 | 0.19 | 0.04 | 1.22 | <.001 |  | 0.67 | 0.13 | 0.42 | 0.93 | <.001 |
| Quintile 4 | 0.87 | 0.23 | 0.40 | 1.33 | <.001 |  | 0.83 | 0.13 | 0.42 | 0.93 | <.001 |
| Quintile 5 | 1.05 | 0.22 | 0.61 | 1.5 | <.001 |  | 0.91 | 0.14 | 0.63 | 1.18 | <.001 |
| Surgery Duration | -0.21 | 0.02 | -0.26 | -0.16 | <.001 |  | -0.15 | 0.02 | -0.18 | -0.12 | <.001 |
| Surgery Urgency |  |  |  |  |  |  |  |  |  |  |  |
| Elective | Ref |  |  |  |  |  |  |  |  |  |  |
| Emergent | -0.73 | 0.19 | -1.11 | -0.34 | <.001 |  | -0.26 | 0.10 | -0.46 | -0.07 | <.01 |
| Total beds | 0.00 | 0.00 | -0.01 | 0.01 | 0.94 |  | 0.00 | 0.00 | -0.01 | 0.01 | 0.70 |
| Preop ICU | -0.37 | 0.27 | -0.91 | 0.17 | 0.17 |  | -0.59 | 0.15 | -0.90 | -0.28 | <.001 |
| Year |  |  |  |  |  |  |  |  |  |  |  |
| 2009-14 | Ref |  |  |  |  |  |  |  |  |  |  |
| Nk ml,12015-19 | 0.49 | 0.19 | 0.12 | 0.87 | <.05 |  | 0.58 | 0.12 | 0.35 | 0.81 | <.001 |
| Teaching hospital | -0.03 | 2.01 | -4.02 | 3.95 | 0.99 |  | 0.08 | 1.62 | -3.13 | 3.30 | 0.96 |
| BMI |  |  |  |  |  |  |  |  |  |  |  |
| <25 | Ref |  |  |  |  |  |  |  |  |  |  |
| 26-30 | -0.05 | 0.14 | -0.33 | 0.23 | 0.70 |  | 0.1 | 0.08 | -0.04 | 0.30 | 0.13 |
| ≥ 31 | -0.31 | 0.16 | -0.63 | 0.02 | 0.06 |  | -0.25 | 0.10 | -0.45 | -0.05 | <.05 |
| Smoking status |  |  |  |  |  |  |  |  |  |  |  |
| Non-smoker | Ref |  |  |  |  |  |  |  |  |  |  |
| Former smoker | 0.08 | 0.12 | -0.15 | 0.32 | 0.48 |  | 0.13 | 0.08 | -0.04 | 0.30 | 0.13 |
| Current smoker | 0.06 | 0.14 | -0.22 | 0.33 | 0.68 |  | -0.09 | 0.09 | -0.27 | 0.09 | 0.34 |
| Charlson CI ≥ 2 | -0.61 | 0.21 | -1.03 | -0.19 | <.01 |  | -0.09 | 0.09 | -0.27 | 0.09 | 0.34 |
| Asthma | -0.22 | 0.12 | -0.45 | 0.02 | 0.07 |  | 0.03 | 0.06 | -0.08 | 0.15 | 0.57 |
| CAD | 0.83 | 0.14 | 0.54 | 1.11 | <.001 |  | 1.00 | 0.06 | 0.88 | 1.12 | <.001 |
| AF | -0.58 | 0.21 | -1.00 | -0.18 | <.01 |  | -0.89 | 0.19 | -1.27 | -0.50 | <.001 |
| PVD | 0.96 | 0.26 | 0.45 | 1.48 | <.001 |  | 0.34 | 0.13 | 0.08 | 0.60 | <.05 |
| Stroke/TIA | 1.41 | 0.55 | 0.32 | 2.49 | <.05 |  | 2.39 | 0.20 | 1.98 | 2.79 | <.001 |
| COPD | -0.86 | 0.18 | -1.21 | -0.51 | <.001 |  | -0.63 | 0.11 | -0.86 | -0.40 | <.001 |
| Hypertension | 0.09 | 0.13 | -0.18 | 0.35 | 0.51 |  | 0.27 | 0.07 | 0.12 | 0.42 | <.001 |
| Kidney disease |  |  |  |  |  |  |  |  |  |  |  |
| Stage 1 | Ref |  |  |  |  |  |  |  |  |  |  |
| Stage 2 | 0.29 | 0.16 | -0.01 | 0.6 | 0.06 |  | 0.42 | 0.12 | 0.19 | 0.42 | <.001 |
| Stage 3 | -0.66 | 0.26 | -1.17 | -0.14 | <.05 |  | -0.04 | 0.26 | -0.56 | 0.48 | 0.87 |
| Stage 4 | -4.33 | 1.09 | -6.49 | -2.16 | <.001 |  | -2.54 | 0.60 | -3.73 | -1.34 | <.001 |
| Stage 5 | -5.49 | 1.53 | -8.53 | -2.44 | <.001 |  | -2.16 | 0.62 | -3.39 | -0.94 | <.001 |
| Diabetes | -0.41 | 0.11 | -0.64 | -0.18 | <.001 |  | -0.28 | 0.07 | -0.42 | -0.14 | <.001 |
| Dementia | -1.68 | 4.56 | -1.08 | 7.43 | 0.72 |  | 0.52 | 1.46 | -2.37 | 3.41 | 0.72 |
| Liver disease | -0.11 | 1.60 | -3.29 | 3.07 | 0.95 |  | 0.61 | 0.66 | -0.69 | 1.91 | 0.36 |
| Cancer | 1.39 | 0.35 | 0.69 | 2.09 | <.001 |  | 1.07 | 0.19 | 0.69 | 1.44 | <.001 |
| LV Function |  |  |  |  |  |  |  |  |  |  |  |
| ≥ 50% | Ref |  |  |  |  |  |  |  |  |  |  |
| 35-49% | -0.13 | 0.15 | -0.43 | 0.17 | 0.39 |  | -0.11 | 0.06 | -0.23 | 0.01 | 0.07 |
| 20-34% | -0.76 | 0.26 | -1.28 | -0.24 | <.01 |  | -0.67 | 0.15 | -0.96 | -0.38 | <.001 |
| <20% | 0.00 | 0.95 | -1.88 | 1.89 | 1 |  | -0.69 | 0.45 | -1.59 | 0.21 | 0.13 |
| Anemia | 1.42 | 0.28 | 0.86 | 1.97 | <.001 |  | 0.89 | 0.20 | 0.49 | 1.20 | <.001 |
| Frailty | -0.98 | 0.14 | -1.26 | -0.69 | <.001 |  | -0.95 | 0.10 | -0.07 | -0.74 | <.001 |

| 1. **Days alive and out of hospital at 180-days** | | | | | | | | | | | |
| --- | --- | --- | --- | --- | --- | --- | --- | --- | --- | --- | --- |
| **Female** |  |  |  |  |  |  | **Males** |  |  |  |  |
| **Variable** | **Parameter estimate (days)** | **St Err** | **95% LCL** | **95% UCL** | **P-value** |  | **Parameter estimate (days)** | **St Err** | **95% LCL** | **95% UCL** | **P-value** |
| Surgery |  |  |  |  |  |  |  |  |  |  |  |
| CABG | Ref |  |  |  |  |  |  |  |  |  |  |
| Non-CABG | -2.02 | 0.43 | -2.87 | -1.16 | <.001 |  | -1.44 | 0.24 | -1.92 | -0.97 | <.001 |
| Combined | -2.08 | 0.33 | -2.73 | -1.44 | <.001 |  | -1.84 | 0.24 | -2.31 | -1.36 | <.001 |
| Age | -0.19 | 0.05 | -0.28 | -0.10 | <.001 |  | -2.02 | 0.03 | -0.27 | -0.14 | <.001 |
| Preop DAH | 0.15 | 0.07 | 0.01 | 0.29 | <.05 |  | 0.07 | 0.05 | -0.03 | 0.16 | 0.19 |
| Surgical volume | 0.00 | 0.00 | -0.02 | 0.00 | 0.23 |  | 0.00 | 0.00 | 0.00 | 0.00 | <.05 |
| Rurality | 0.40 | 0.26 | -0.12 | 0.91 | 0.13 |  | 0.14 | 0.15 | -0.16 | 0.44 | 0.35 |
| Income Quintile |  |  |  |  |  |  |  |  |  |  |  |
| Quintile 1 | Ref |  |  |  |  |  |  |  |  |  |  |
| Quintile 2 | 1.89 | 0.40 | 1.10 | 2.68 | <.001 |  | 0.93 | 0.28 | 0.38 | 1.49 | <.01 |
| Quintile 3 | 2.16 | 0.44 | 1.29 | 3.02 | <.001 |  | 1.12 | 0.28 | 0.57 | 1.67 | <.001 |
| Quintile 4 | 2.34 | 0.42 | 1.51 | 3.16 | <.001 |  | 1.03 | 0.29 | 0,46 | 1.61 | <.001 |
| Quintile 5 | 2.62 | 0.43 | 1.78 | 3.46 | <.001 |  | 1.40 | 0.29 | 0.83 | 1.96 | <.001 |
| Surgery Duration | -0.39 | 0.07 | -0.53 | -0.26 | <.001 |  | -0.19 | 0.03 | -0.25 | -0.12 | <.001 |
| Surgery Urgency |  |  |  |  |  |  |  |  |  |  |  |
| Elective | Ref |  |  |  |  |  |  |  |  |  |  |
| Emergent | 0.02 | 0.37 | -0.20 | 0.75 | 0.95 |  | -0.20 | 0.23 | -0.66 | 0.26 | 0.40 |
| Total beds | 0.00 | 0.01 | -0.02 | 0.02 | 0.72 |  | 0.00 | 0.00 | -0.01 | 0.01 | 0.95 |
| Preop ICU | -0.81 | 0.54 | -1.88 | 0.27 | 0.14 |  | -0.49 | 0.24 | -0.97 | -0.01 | <.05 |
| Year |  |  |  |  |  |  |  |  |  |  |  |
| 2009-14 | Ref |  |  |  |  |  |  |  |  |  |  |
| 2015-19 | 0.68 | 0.30 | 0.09 | 1.27 | 0.03 |  | 0.83 | 0.19 | 0.45 | 1.21 | <.001 |
| Teaching hospital | 1.05 | 4.22 | -7.32 | 9.42 | 0.8 |  | 0.02 | 2.53 | -5.00 | 5.04 | 0.99 |
| BMI |  |  |  |  |  |  |  |  |  |  |  |
| <25 | Ref |  |  |  |  |  |  |  |  |  |  |
| 26-30 | 0.49 | 0.27 | -0.05 | 1.03 | 0.08 |  | 0.23 | 0.14 | -0.05 | 0.51 | 0.11 |
| ≥ 31 | 0.27 | 0.30 | -0.32 | 0.87 | 0.36 |  | -0.33 | 1.71 | -0.67 | 0.01 | 0.05 |
| Smoking status |  |  |  |  |  |  |  |  |  |  |  |
| Non-smoker | Ref |  |  |  |  |  |  |  |  |  |  |
| Former smoker | -0.02 | 0.19 | -0.39 | 0.35 | 0.91 |  | 0.24 | 0.12 | -0.01 | 0.49 | 0.06 |
| Current smoker | -0.03 | 0.41 | -0.85 | 0.79 | 0.94 |  | -0.35 | 0.19 | -0.72 | 0.02 | 0.06 |
| Charlson CI ≥ 2 | -1.20 | 0.33 | -1.84 | -0.55 | <.001 |  | -0.26 | 0.15 | -0.56 | 0.05 | 0.10 |
| Asthma | -0.35 | 0.20 | -0.75 | 0.05 | 0.09 |  | -0.24 | 0.13 | -0.50 | 0.01 | 0.06 |
| CAD | 1.80 | 0.24 | 1.32 | 2.28 | <.001 |  | 1.35 | 0.14 | 1.07 | 1.64 | <.001 |
| AF | 0.22 | 0.32 | -0.42 | 0.86 | 0.49 |  | -0.77 | 0.24 | -1.25 | -0.29 | <.01 |
| PVD | 2.12 | 0.41 | 1.30 | 2.94 | <.001 |  | 0.72 | 0.18 | 0.36 | 1.07 | <.001 |
| Stroke/TIA | 5.09 | 0.72 | 3.65 | 6.53 | <.001 |  | 1.10 | 0.35 | 0.41 | 1.79 | <.01 |
| COPD | -0.81 | 0.30 | -1.40 | -0.2 | <.01 |  | -0.81 | 0.18 | -1.18 | -0.45 | <.001 |
| Hypertension | 0.47 | 0.27 | -0.07 | 1.01 | 0.08 |  | 0.71 | 0.15 | 0.41 | 1.01 | <.001 |
| Kidney disease |  |  |  |  |  |  |  |  |  |  |  |
| Stage 1 | Ref |  |  |  |  |  |  |  |  |  |  |
| Stage 2 | 0.91 | 0.38 | 0.16 | 1.66 | <.05 |  | 1.33 | 0.24 | 0.85 | 1.81 | <.001 |
| Stage 3 | 0.47 | 0.50 | -0.52 | 1.47 | 0.35 |  | 0.66 | 0.45 | -0.23 | 1.54 | 0.14 |
| Stage 4 | -4.43 | 1.23 | -6.87 | -1.99 | <.001 |  | -0.98 | 0.92 | -2.80 | 0.85 | 0.29 |
| Stage 5 | -4.27 | 2.54 | -9.32 | 0.79 | 0.1 |  | -0.33 | 0.98 | -2.27 | 1.62 | 0.74 |
| Diabetes | -0.48 | 0.23 | -0.93 | -0.04 | <.05 |  | -0.33 | 0.98 | -2.27 | 1.62 | 0.74 |
| Dementia | 8.17 | 4.14 | -4.88 | 16.39 | 0.05 |  | 14.91 | 3.13 | 8.69 | 21.13 | <.001 |
| Liver disease | -0.17 | 1.76 | -3.66 | 3.32 | 0.92 |  | 3.52 | 1.05 | 1.43 | 5.61 | <.01 |
| Cancer | 3.73 | 0.46 | 2.81 | 4.65 | <.001 |  | 2.52 | 0.37 | 1.79 | 3.25 | <.001 |
| LV Function |  |  |  |  |  |  |  |  |  |  |  |
| ≥ 50% | Ref |  |  |  |  |  |  |  |  |  |  |
| 35-49% | 0.64 | 0.19 | 0.25 | 1.03 | <.01 |  | -0.2 | 0.11 | -0.41 | 0.01 | 0.06 |
| 20-34% | 0.24 | 0.42 | -0.59 | 1.07 | 0.57 |  | -0.49 | 0.22 | -0.92 | -0.05 | <.05 |
| <20% | 4.13 | 0.99 | 2.17 | 6.1 | <.001 |  | 0.66 | 0.49 | -0.32 | 1.64 | 0.19 |
| Anemia | 1.80 | 0.47 | 0.91 | 2.68 | <.001 |  | 1.48 | 0.29 | 0.90 | 2.06 | <.001 |
| Frailty | -1.13 | 0.19 | -1.50 | -0.76 | <.001 |  | -1.04 | 0.12 | -1.27 | -0.83 | <.001 |

*AF atrial fibrillation; CAD coronary artery disease; Charlson CI Charlson comorbidity index; COPD chronic obstructive pulmonary disease; DAH days alive and out of hospital; LV left ventricular; PVD peripheral vascular disease*

*^a^Surgery Duration per 10 min*

**Table 4. Summary of analyses with interaction variable.**

|  | Co-efficient | P-value* |
| --- | --- | --- |
| Male patient: Teaching vs. community hospital | 0.53 | 0.61 |
| Female patient: Teaching vs. community hospital | 0.63 |  |
| Male patient: Complications vs. no complications | -2.18 | 0.43 |
| Female patient: Complications vs. no complications | -2.45 |  |
| Male patient: Isolated non-CABG vs. Isolated CABG | -0.87 | 0.01 |
| Female patient: Isolated non-CABG vs. Isolated CABG | -0.54 |  |
| Male patient: Combination surgery vs. Isolated CABG | -1.24 | 0.95 |
| Female patient: Combination surgery vs. Isolated CABG | -1.25 |  |
| Male patient: Duration of surgery | -0.10 | <.001 |
| Female patient: Duration of surgery | -0.15 |  |

**p-value for interaction variable*

*Interaction studied were between sex and surgical group, complications, duration if surgery and hospital teaching status.*

**Table 5.** **Characteristics of male and female patients above and below the 10^th^ percentile.**

| **Variable** | **> 10th percentile** | | **≤ 10th percentile** | | **Total** | | **Standardized difference^a^** | | **P-value^a^** | |
| --- | --- | --- | --- | --- | --- | --- | --- | --- | --- | --- |
|  | **Female** | **Male** | **Female** | **Male** | **Female** | **Male** | **Female** | **Male** | **Female** | **Male** |
|  | **N=20,110** | **N=58,402** | **N=2,160** | **N=6,307** | **N=22,270** | **N=64,709** |  |  |  |  |
| **Patient Factors** | | | | | | | | | | |
| Age | 69 (61-76) | 66 (59-73) | 74 (66-79) | 73 (65-79) | 70 (62-76) | 67 (59-74) | 0.37 | 0.53 | <.001 | <.001 |
| Atrial fibrillation | 2,735 (13.6%) | 5,519 (9.5%) | 596 (27.6%) | 1,538 (24.4%) | 3,331 (15.0%) | 7,057 (10.9%) | 0.35 | 0.41 | <.001 | <.001 |
| Anemia | 1,836 (9.1%) | 2,758 (4.7%) | 457 (21.2%) | 965 (15.3%) | 2,293 (10.3%) | 3,723 (5.8%) | 0.34 | 0.36 | <.001 | <.001 |
| Asthma | 3,722 (18.5%) | 6,077 (10.4%) | 496 (23.0%) | 863 (13.7%) | 4,218 (18.9%) | 6,940 (10.7%) | 0.11 | 0.1 | <.001 | <.001 |
| CAD | 9,581 (47.6%) | 30,916 (52.9%) | 1,356 (62.8%) | 4,208 (66.7%) | 10,937 (49.1%) | 35,124 (54.3%) | 0.31 | 0.28 | <.001 | <.001 |
| BMI |  |  |  |  |  |  |  |  |  |  |
| ≤ 25 | 5,993 (29.8%) | 13,805 (23.6%) | 686 (31.8%) | 1,757 (27.9%) | 6,679 (30.0%) | 15,562 (24.0%) | 0.04 | 0.1 | <.001 | <.001 |
| 26-30 | 6,445 (32.0%) | 24,764 (42.4%) | 621 (28.8%) | 2,291 (36.3%) | 7,066 (31.7%) | 27,055 (41.8%) | 0.07 | 0.12 | 0.006 | <.001 |
| > 31 | 7,672 (38.2%) | 19,833 (34.0%) | 853 (39.5%) | 2,259 (35.8%) | 8,525 (38.3%) | 22,092 (34.1%) | 0.03 | 0.04 | <.001 | <.001 |
| COPD | 2,218 (11.0%) | 4,908 (8.4%) | 429 (19.9%) | 1,271 (20.2%) | 2,647 (11.9%) | 6,179 (9.5%) | 0.25 | 0.34 | <.001 | <.001 |
| Stroke/TIA | 488 (2.4%) | 1,251 (2.1%) | 125 (5.8%) | 361 (5.7%) | 613 (2.8%) | 1,612 (2.5%) | 0.17 | 0.19 | 0.004 | <.001 |
| Dementia | 33 (0.2%) | 78 (0.1%) | 26 (1.2%) | 49 (0.8%) | 59 (0.3%) | 127 (0.2%) | 0.13 | 0.1 | 0.027 | 0.004 |
| Diabetes | 8,745 (43.5%) | 24,897 (42.6%) | 1,140 (52.8%) | 3,312 (52.5%) | 9,885 (44.4%) | 28,209 (43.6%) | 0.19 | 0.2 | <.001 | 0.027 |
| Smoking status |  |  |  |  |  |  |  |  |  |  |
| Never | 12,118 (60.3%) | 11,009 (18.9%) | 1,340 (62.0%) | 1,204 (19.1%) | 13,458 (60.4%) | 12,213 (18.9%) | 0.04 | 0.01 | <.001 | <.001 |
| Former | 5,021 (25.0%) | 22,827 (39.1%) | 502 (23.2%) | 2,627 (41.7%) | 5,523 (24.8%) | 25,454 (39.3%) | 0.04 | 0.05 | <.001 | <.001 |
| Current | 2,971 (14.8%) | 24,566 (42.1%) | 318 (14.7%) | 2,476 (39.3%) | 3,289 (14.8%) | 27,042 (41.8%) | 0 | 0.06 | <.001 | <.001 |
| Hypertension | 16,963 (84.4%) | 48,576 (83.2%) | 1,957 (90.6%) | 5,544 (87.9%) | 18,920 (85.0%) | 54,120 (83.6%) | 0.19 | 0.13 | <.001 | <.001 |
| Chronic liver disease | 100 (0.5%) | 334 (0.6%) | 48 (2.2%) | 152 (2.4%) | 148 (0.7%) | 486 (0.8%) | 0.15 | 0.15 | 0.785 | <.001 |
| Myocardial infarction | 4,487 (22.3%) | 14,797 (25.3%) | 747 (34.6%) | 2,139 (33.9%) | 5,234 (23.5%) | 16,936 (26.2%) | 0.27 | 0.19 | <.001 | 0.785 |
| PVD | 1,237 (6.2%) | 3,414 (5.8%) | 220 (10.2%) | 683 (10.8%) | 1,457 (6.5%) | 4,097 (6.3%) | 0.15 | 0.18 | 0.224 | <.001 |
| Frailty | 1 (0-4) | 1 (0-3) | 8 (4-13) | 4 (2-9) | 2 (0-5) | 2 (0-4) | 1.33 | 0.88 | <.001 | 0.224 |
| CCI ≥ 2 | 5,720 (28.4%) | 15,084 (25.8%) | 1,231 (57.0%) | 3,325 (52.7%) | 6,951 (31.2%) | 18,409 (28.4%) | 0.6 | 0.57 | <.001 | <.001 |
| CKD |  |  |  |  |  |  |  |  |  |  |
| 1 | 5,385 (26.8%) | 20,076 (34.4%) | 197 (9.1%) | 835 (13.2%) | 5,582 (25.1%) | 20,911 (32.3%) | 0.47 | 0.51 | <.001 | <.001 |
| 2 | 9,279 (46.1%) | 27,156 (46.5%) | 705 (32.6%) | 2,404 (38.1%) | 9,984 (44.8%) | 29,560 (45.7%) | 0.28 | 0.17 | <.001 | <.001 |
| 3 | 4,670 (23.2%) | 9,586 (16.4%) | 870 (40.3%) | 2,188 (34.7%) | 5,540 (24.9%) | 11,774 (18.2%) | 0.37 | 0.43 | <.001 | <.001 |
| 4 | 576 (2.9%) | 1,066 (1.8%) | 288 (13.3%) | 602 (9.5%) | 864 (3.9%) | 1,668 (2.6%) | 0.39 | 0.34 | <.001 | <.001 |
| 5 | 200 (1.0%) | 518 (0.9%) | 100 (4.6%) | 278 (4.4%) | 300 (1.3%) | 796 (1.2%) | 0.22 | 0.22 | <.001 | <.001 |
| LV function |  |  |  |  |  |  |  |  |  |  |
| <20% | 131 (0.7%) | 832 (1.4%) | 42 (1.9%) | 281 (4.5%) | 173 (0.8%) | 1,113 (1.7%) | 0.11 | 0.18 | <.001 | <.001 |
| 20% - 34% | 1,054 (5.2%) | 4,610 (7.9%) | 253 (11.7%) | 961 (15.2%) | 1,307 (5.9%) | 5,571 (8.6%) | 0.23 | 0.23 | <.001 | <.001 |
| 35% - 49% | 2,977 (14.8%) | 12,191 (20.9%) | 435 (20.1%) | 1,581 (25.1%) | 3,412 (15.3%) | 13,772 (21.3%) | 0.14 | 0.1 | <.001 | <.001 |
| ≥ 50% | 15,948 (79.3%) | 40,769 (69.8%) | 1,430 (66.2%) | 3,484 (55.2%) | 17,378 (78.0%) | 44,253 (68.4%) | 0.3 | 0.3 | <.001 | <.001 |
| ICU pre-surgery | 11,570 (57.5%) | 34,396 (58.9%) | 1,285 (59.5%) | 3,888 (61.6%) | 12,855 (57.7%) | 38,284 (59.2%) | 0.04 | 0.06 | 0.08 | <.001 |
| Income Quintile |  |  |  |  |  |  |  |  |  | <.001 |
| 1 | 4,234 (21.1%) | 9,853 (16.9%) | 570 (26.4%) | 1,359 (21.5%) | 4,804 (21.6%) | 11,212 (17.3%) | 0.13 | 0.12 | <.001 | <.001 |
| 2 | 4,235 (21.1%) | 11,529 (19.7%) | 471 (21.8%) | 1,386 (22.0%) | 4,727 (21.2%) | 12,915 (20.0%) | 0.02 | 0.06 | <.001 | <.001 |
| 3 | 4,119 (20.5%) | 12,145 (20.8%) | 432 (20.0%) | 1,240 (19.7%) | 4,551 (20.4%) | 13,385 (20.7%) | 0.01 | 0.03 | <.001 | <.001 |
| 4 | 3,785 (18.8%) | 12,358 (21.2%) | 383 (17.7%) | 1,179 (18.7%) | 4,168 (18.7%) | 13,537 (20.9%) | 0.03 | 0.06 | <.001 | <.001 |
| 5 | 3,716 (18.5%) | 12,517 (21.4%) | 304 (14.1%) | 1,143 (18.1%) | 4,020 (18.1%) | 13,660 (21.1%) | 0.12 | 0.08 | <.001 | <.001 |
| Preop DAH (3m – 1d) | 90 (83-92) | 90 (84-92) | 84 (75-90) | 85 (78-91) | 89 (83-92) | 90 (83-92) | 0.6 | 0.48 | <.001 | <.001 |
| **Surgery factors** | | | | | | | | | | |
| Isolated CABG | 10,037 (49.9%) | 40,532 (69.4%) | 809 (37.5%) | 3,026 (48.0%) | 10,846 (48.7%) | 43,558 (67.3%) | 0.25 | 0.45 | <.001 | <.001 |
| Combined Surgery | 5,006 (24.9%) | 10,580 (18.1%) | 925 (42.8%) | 2,442 (38.7%) | 5,931 (26.6%) | 13,022 (20.1%) | 0.39 | 0.47 | <.001 | <.001 |
| Single non-CABG | 5,067 (25.2%) | 7,290 (12.5%) | 426 (19.7%) | 839 (13.3%) | 5,493 (24.7%) | 8,129 (12.6%) | 0.13 | 0.02 | <.001 | <.001 |
| **Hospital factors** | | | | | | | | | | |
| Teaching hospital | 12,236 (60.8%) | 34,598 (59.2%) | 1,283 (59.4%) | 3,793 (60.1%) | 13,519 (60.7%) | 38,391 (59.3%) | 0.03 | 0.02 | 0.191 | 0.191 |
| Total Beds | 416 (291-722) | 416 (291-527) | 360 (287-513) | 3,793 (60.1%) | 403 (291-555) | 38,391 (59.3%) | 0.14 | 0.02 | <.001 | <.001 |
| **Outcomes** |  |  |  |  |  |  |  |  |  |  |
| DAH_30_ | 22 (19-24) | 24 (22-25) | 0 (0-1) | 3 (0-9) | 22 (17-24) | 23 (20-25) | 2.72 | **2.73** | <.001 | <.001 |
| DAH_90_ | 82 (77-84) | 84 (81-85) | 34 (0-55) | 57 (20-67 | 81 (75-84) | 83 (79-85) | 2.61 | 2.65 | <.001 | **<.001** |
| DAH_180_ | 171 (166-174) | 173 (170-175) | 114 (0-143) | 144 (86-156) | 171 (163-174) | 173 (168-175) | 2.55 | 2.58 | <.001 | <.001 |
| 30-day mortality | 0 (0.0%) | 0 (0.0%) | 7 (0.3%) | 8 (0.1%) | 7 (0.0%) | 9 (0.0%) | 0.08 | 0.05 | <.001 | <.001 |
| Major complications | 3,874 (19.3%) | 8,367 (14.3%) | 818 (37.9%) | 2,498 (39.6%) | 4,692 (21.1%) | 10,865 (16.8%) | 0.42 | 0.59 | <.001 | <.001 |
| Postop LOS | 7 (5-9) | 6 (5-7) | 25 (12-38) | 19 (11-28) | 7 (6-10) | 6 (5-8) | 1.16 | 1.55 | <.001 | <.001 |

**Table 6. Days alive and out of hospital at 30 and 90 days for subtypes of isolated non-CABG and combined surgery stratified by patient sex.**

| **Single non-CABG Surgery** | **DAH_30_** | | | | | | **DAH_90_** | | | | | |
| --- | --- | --- | --- | --- | --- | --- | --- | --- | --- | --- | --- | --- |
|  | **Female** | | | **Male** | | | **Female** | | | **Male** | | |
|  | **N** | **Median** | **IQR** | **N** | **Median** | **IQR** | **N** | **Median** | **IQR** | **N** | **Median** | **IQR** |
| Aortic valve | 3346 | 22 | 18-24 | 5109 | 23 | 20-25 | 3346 | 82 | 76-84 | 5109 | 83 | 79-85 |
| Mitral valve | 1846 | 22 | 17-24 | 2598 | 23 | 20-25 | 1846 | 81 | 76-84 | 2598 | 83 | 79-85 |
| Pulmonic and tricuspid valve | 309 | 20 | 8-23 | 340 | 22 | 15-24 | 309 | 80 | 64-83 | 340 | 81 | 73-84 |
| Aneuryesectomy or aortic only procedure | 60 | 18 | 9.5-21 | 144 | 22 | 17-24 | 60 | 77 | 70-81 | 144 | 81 | 76-84 |
| **TOTAL** | **5561** | **22** | **17-24** | **8191** | **23** | **20-25** | **5561** | **82** | **76-84** | **8191** | **83** | **70-85** |

| **Combined Procedures** | **DAH_30_** | | | | | | **DAH_90_** | | | | | |
| --- | --- | --- | --- | --- | --- | --- | --- | --- | --- | --- | --- | --- |
|  | **Female** | | | **Male** | | | **Female** | | | **Male** | | |
|  | **N** | **Median** | **IQR** | **N** | **Median** | **IQR** | **N** | **Median** | **IQR** | **N** | **Median** | **IQR** |
| CABG/valve^a^ | 2742 | 19 | 11-23 | 7873 | 21 | 15-24 | 2742 | 78 | 67-82 | 7873 | 81 | 73-84 |
| 2 valves | 1296 | 19 | 12-23 | 1220 | 21 | 14-23 | 1296 | 78 | 69-82 | 1220 | 80 | 71-83 |
| ≥3 procedures^b^ | 754 | 16 | 3-21 | 1432 | 19 | 10-23 | 754 | 75 | 58-81 | 1432 | 78 | 67-82 |
| Other^c^ | 1278 | 22 | 16-24 | 2761 | 23 | 18-24 | 1278 | 81 | 75-81 | 2761 | 82 | 77-84 |
| **TOTAL** | **6070** | **19** | **12-23** | **13286** | **21** | **15-24** | **6070** | **78** | **68-83** | **13286** | **81** | **73-84** |

CABG/valve^a^  includes few procedures with aortic or aneurysectomy surgery

≥3 procedures^b^ predominantly includes triple valve surgery, quadruple valve surgeries, few patients undergoing double valve with third procedure such as CABG or aortic surgery.

Other^c^ includes valve with single non-CABG surgery such as aortic or aneurysectomy.

**Table 7. Risk adjusted effects of patient, surgical and hospital factors on days at alive and out of hospital (DAH) at 30-days for women and men undergoing A) single non-CABG and B) combined surgical procedures.**

| 1. **Single non-CABG procedures** | | | | | | | | | | | |
| --- | --- | --- | --- | --- | --- | --- | --- | --- | --- | --- | --- |
| **Female** |  |  |  |  |  |  | **Males** |  |  |  |  |
| **Variable** | **Parameter estimate (days)** | **St Err** | **95% LCL** | **95% UCL** | **P-value** |  | **Parameter estimate (days)** | **St Err** | **95% LCL** | **95% UCL** | **P-value** |
| Surgery |  |  |  |  |  |  |  |  |  |  |  |
| AV | Ref |  |  |  |  |  |  |  |  |  |  |
| Aorta/Aneurysmectomy | -0.79 | 1.12 | -3.00 | 1.43 | 0.48 |  | 0.10 | 0.34 | -0.58 | 0.79 | 0.76 |
| MV | -0.44 | 0.29 | -1.00 | 0.14 | 0.13 |  | -0.22 | 0.17 | -0.56 | 0.11 | 0.19 |
| TV/PV | -1.64 | 0.95 | -3.52 | 0.23 | 0.09 |  | -0.95 | 0.64 | -2.22 | 0.32 | 0.14 |
| Age | -0.04 | 0.01 | -0.06 | -0.03 | <.001 |  | -0.40 | 0.01 | -0.06 | -0.02 | <.001 |
| Preop DAH | 0.08 | 0.02 | 0.05 | 0.11 | <.001 |  | 0.05 | 0.01 | 0.03 | 0.06 | <.001 |
| Surgical volume | 0.00 | 0.00 | 0.00 | 0.00 | 0.90 |  | 0.00 | 0.00 | 0.00 | 0.00 | 0.73 |
| Rurality | 0.15 | 0.19 | -0.22 | 0.52 | 0.44 |  | 0.09 | 0.09 | -0.09 | 0.28 | 0.32 |
| Income Quintile |  |  |  |  |  |  |  |  |  |  |  |
| Quintile 1 | Ref |  |  |  |  |  |  |  |  |  |  |
| Quintile 2 | 0.28 | 0.18 | -0.09 | 0.65 | 0.14 |  | 0.19 | 0.15 | -0.10 | 0.48 | 0.19 |
| Quintile 3 | 0.25 | 0.19 | -0.13 | 0.64 | 0.20 |  | 0.25 | 0.16 | -0.78 | 0.57 | 0.13 |
| Quintile 4 | 0.25 | 0.23 | -0.21 | 0.70 | 0.29 |  | 0.37 | 0.15 | 0.08 | 0.66 | 0.01 |
| Quintile 5 | 0.75 | 0.16 | 0.43 | 1.07 | <.001 |  | 0.50 | 0.15 | 0.20 | 0.80 | <.01 |
| Surgery Duration | -0.12 | 0.01 | -0.14 | -0.09 | <.001 |  | -0.08 | 0.01 | -0.10 | -0.06 | <.001 |
| Surgery Urgency |  |  |  |  |  |  |  |  |  |  |  |
| Elective | Ref |  |  |  |  |  |  |  |  |  |  |
| Emergent | -1.28 | 0.28 | -1.84 | -0.72 | <.001 |  | -1.00 | 0.23 | -1.46 | -0.55 | <.001 |
| Total beds | 0.00 | 0.00 | 0.00 | 0.00 | 0.85 |  | 0.00 | 0.00 | 0.00 | 0.00 | 0.28 |
| Preop ICU | -0.14 | 0.15 | -0.44 | 0.17 | 0.38 |  | -0.27 | 0.17 | -0.61 | 0.07 | 0.11 |
| Year |  |  |  |  |  |  |  |  |  |  |  |
| 2009-14 | Ref |  |  |  |  |  |  |  |  |  |  |
| 2015-19 | 0.31 | 0.25 | -0.19 | 0.80 | 0.22 |  | 0.62 | 0.10 | 0.41 | 0.82 | <.001 |
| Teaching hospital | 0.26 | 0.65 | -1.03 | 1.54 | 0.69 |  | 0.31 | 0.44 | -0.56 | 1.18 | 0.48 |
| BMI |  |  |  |  |  |  |  |  |  |  |  |
| <25 | Ref |  |  |  |  |  |  |  |  |  |  |
| 26-30 | -0.05 | -0.17 | -0.39 | 0.28 | 0.76 |  | 0.11 | 0.10 | -0.09 | 0.31 | 0.29 |
| ≥ 31 | -0.17 | 0.14 | -0.44 | 0.11 | 0.23 |  | -0.20 | 0.15 | -0.50 | 0.10 | 0.19 |
| Smoking status |  |  |  |  |  |  |  |  |  |  |  |
| Non-smoker | Ref |  |  |  |  |  |  |  |  |  |  |
| Former smoker | -0.18 | 0.12 | -0.06 | 0.42 | 0.14 |  | -0.02 | 0.08 | -0.17 | 0.13 | 0.79 |
| Current smoker | -0.08 | 0.23 | -1.08 | -0.16 | <.01 |  | -0.14 | 0.16 | -0.37 | 0.09 | 0.23 |
| Charlson CI ≥ 2 | -0.62 | 0.23 | -1.08 | -0.16 | <.01 |  | -0.15 | 0.17 | -0.38 | 0.08 | 0.21 |
| Asthma | -0.02 | 0.09 | -0.20 | 0.16 | 0.86 |  | -0.25 | 0.14 | -0.53 | 0.03 | 0.08 |
| CAD | 0.40 | 0.21 | -0.26 | 0.82 | 0.07 |  | 0.32 | 0.13 | 0.05 | 0.59 | <.05 |
| AF | -0.44 | -0.13 | -0.70 | -0.18 | <.01 |  | -0.59 | 0.15 | -0.88 | -0.31 | <.001 |
| PVD | -0.39 | 0.50 | -1.39 | 0.61 | 0.44 |  | 0.19 | 0.17 | -0.16 | 0.53 | 0.28 |
| Stroke/TIA | 1.84 | 0.56 | 0.73 | 2.96 | <.01 |  | 0.70 | 0.50 | -0.30 | 1.69 | 0.17 |
| COPD | -0.51 | 0.20 | -0.91 | -0.11 | <.05 |  | -0.21 | 0.09 | -0.39 | -0.02 | <.05 |
| Hypertension | -0.11 | 0.11 | -0.33 | 0.10 | 0.30 |  | 0.23 | 0.11 | 0..1 | 0.45 | <.05 |
| Kidney disease |  |  |  |  |  |  |  |  |  |  |  |
| Stage 1 | Ref |  |  |  |  |  |  |  |  |  |  |
| Stage 2 | -0.12 | 0.12 | -0.35 | 0.10 | 0.28 |  | -0.09 | 0.15 | -0.39 | 0.21 | 0.54 |
| Stage 3 | -0.58 | 0.23 | -1.04 | -0.11 | <.05 |  | -0.59 | 0.24 | -1.06 | -0.12 | <.05 |
| Stage 4 | -3.40 | 0.73 | -4.85 | -1.96 | <.001 |  | -3.17 | 1.06 | -5.28 | -1.06 | <.01 |
| Stage 5 | -4.30 | 1.87 | -8.01 | -0.59 | <.05 |  | -2.08 | 1.49 | -5.03 | 0.87 | 0.16 |
| Diabetes | -0.21 | 0.15 | -0.51 | 0.10 | 0.19 |  | -0.19 | 0.14 | -0.45 | 0.07 | 0.15 |
| Dementia | -4.04 | 3.46 | -1.09 | 2.82 | 0.25 |  | 3.27 | 1.23 | 0.08 | 5.72 | <.01 |
| Liver disease | 0.26 | 2.14 | -4.00 | 4.52 | 0.90 |  | -0.72 | 1.36 | -0.34 | 1.98 | 0.6 |
| Cancer | 0.81 | 0.29 | 0.23 | 1.40 | <.01 |  | 0.24 | 0.27 | -0.30 | 0.78 | 0.38 |
| LV Function |  |  |  |  |  |  |  |  |  |  |  |
| ≥ 50% | Ref |  |  |  |  |  |  |  |  |  |  |
| 35-49% | 0.49 | 0.18 | 0.13 | 0.86 | <.01 |  | 0.01 | 0.12 | -0.26 | 0.28 | 0.94 |
| 20-34% | 0.17 | 0.55 | -0.93 | 1.26 | 0.76 |  | -0.14 | 0.24 | -0.61 | 0.33 | 0.55 |
| <20% | 1.22 | 2.30 | -3.35 | 5.79 | 0.60 |  | -3.01 | 2.35 | -7.67 | 1.64 | 0.20 |
| Anemia | 0.51 | 0.21 | 0.08 | 0.92 | <.05 |  | -0.67 | 0.07 | -0.80 | -0.54 | <.001 |
| Frailty | -0.70 | 0.09 | -0.87 | -0.53 | <.001 |  | -0.67 | 0.07 | -0.80 | -0.54 | <.001 |
| Major complications | -2.03 | 0.62 | -3.25 | -0.80 | <.01 |  | -1.74 | 0.37 | -2.47 | -1.99 | <.001 |

| **B. Combined procedures** | | | | | | | | | | | |
| --- | --- | --- | --- | --- | --- | --- | --- | --- | --- | --- | --- |
| **Female** |  |  |  |  |  |  | **Males** |  |  |  |  |
| **Variable** | **Parameter estimate (days)** | **St Err** | **95% LCL** | **95% UCL** | **P-value** |  | **Parameter estimate (days)** | **St Err** | **95% LCL** | **95% UCL** | **P-value** |
| Surgery |  |  |  |  |  |  |  |  |  |  |  |
| CABG/valve | Ref |  |  |  |  |  |  |  |  |  |  |
| 2 valves | -0.98 | 0.31 | -1.59 | -0.38 | <.01 |  | -0.88 | 0.24 | -1.36 | -0.41 | <.001 |
| ≥ 3 procedures | -1.36 | 0.30 | -1.95 | -0.77 | <.001 |  | -0.78 | 0.24 | -1.25 | -0.32 | <.01 |
| Other | -0.13 | 0.29 | -0.70 | 0.45 | 0.67 |  | -0.42 | 0.18 | -0.77 | -0.06 | <.05 |
| Age | -0.08 | 0.02 | -0.11 | -0.05 | <.001 |  | -0.76 | 0.02 | -0.11 | -0.04 | <.001 |
| Preop DAH | 0.05 | 0.01 | 0.03 | 0.08 | <.001 |  | 0.00 | 0.01 | -0.01 | 0.02 | 0.72 |
| Surgical volume | 0.00 | 0.00 | 0.00 | 0.00 | 0.79 |  | 0.00 | 0.00 | 0.00 | 0.00 | 0.6 |
| Rurality | 0.05 | 0.18 | -0.31 | 0.41 | 0.79 |  | 0.09 | 0.10 | -0.11 | 0.29 | 0.37 |
| Income Quintile |  |  |  |  |  |  |  |  |  |  |  |
| Quintile 1 | Ref |  |  |  |  |  |  |  |  |  |  |
| Quintile 2 | 0.75 | 0.20 | 0.35 | 1.15 | <.001 |  | -0.22 | 0.16 | -0.53 | 0.10 | 0.17 |
| Quintile 3 | 0.42 | 0.24 | -0.06 | 0.89 | 0.08 |  | 0.23 | 0.14 | -0.54 | 0.51 | 0.11 |
| Quintile 4 | 0.52 | 0.29 | -0.05 | 1.09 | 0.07 |  | 0.33 | 0.10 | 0.19 | 0.54 | <.01 |
| Quintile 5 | 0.83 | 0.40 | 0.05 | 1.62 | <.05 |  | 0.46 | 0.14 | 0.19 | 0.74 | <.01 |
| Surgery Duration | -0.18 | 0.02 | -0.21 | -0.15 | <.001 |  | -0.13 | 0.01 | -0.15 | -0.12 | <.001 |
| Surgery Urgency |  |  |  |  |  |  |  |  |  |  |  |
| Elective | Ref |  |  |  |  |  |  |  |  |  |  |
| Emergent | -0.97 | 0.29 | -1.55 | -0.39 | <.01 |  | -1.27 | 0.26 | -1.78 | -0.75 | <.001 |
| Total beds | 0.00 | 0.00 | -0.01 | 0..00 | 0.65 |  | 0.00 | 0.00 | 0.00 | 0.00 | 0.25 |
| Preop ICU | -0.50 | 0.31 | -1.12 | 0.12 | 0.11 |  | -0.32 | 0.18 | -0.68 | 0.04 | 0.08 |
| Year |  |  |  |  |  |  |  |  |  |  |  |
| 2009-14 | Ref |  |  |  |  |  |  |  |  |  |  |
| 2015-19 | 0.69 | 0.31 | 0.08 | 1.31 | <.05 |  | 0.50 | 0.13 | 0.24 | 0.77 | <.001 |
| Teaching hospital | 0.10 | 0.84 | -0.71 | 2.64 | 0.26 |  | 0.34 | 0.48 | -0.61 | 1.30 | 0.48 |
| BMI |  |  |  |  |  |  |  |  |  |  |  |
| <25 | Ref |  |  |  |  |  |  |  |  |  |  |
| 26-30 | 0.13 | 0.21 | -0.27 | 0.53 | 0.53 |  | 0.29 | 0.14 | 0.01 | 0.56 | <.05 |
| ≥ 31 | -0.19 | 0.22 | -0.62 | 0.25 | 0.40 |  | -0.75 | 0.18 | -0.43 | 0.28 | 0.68 |
| Smoking status |  |  |  |  |  |  |  |  |  |  |  |
| Non-smoker | Ref |  |  |  |  |  |  |  |  |  |  |
| Former smoker | 0.40 | 0.19 | 0.02 | 0.79 | <.05 |  | 0.15 | 0.14 | -0.12 | 0.42 | 0.27 |
| Current smoker | 0.55 | 0.36 | -0.16 | 1.26 | 0.13 |  | 0.17 | 0.10 | -0.02 | 0.36 | 0.08 |
| Charlson CI ≥ 2 | -1.05 | 0.35 | -1.75 | -0.36 | 0.37 |  | -0.35 | 0.13 | -0.60 | -0.09 | <.01 |
| Asthma | -0.29 | 0.33 | -0.94 | 0.36 | 0.37 |  | -0.32 | 0.14 | -0.60 | -0.04 | <.05 |
| CAD | 0.28 | 0.27 | -0.26 | 0.81 | 0.30 |  | 0.55 | 0.16 | 0.23 | 0.86 | <.001 |
| AF | -0.34 | 0.19 | -0.72 | 0.03 | 0.07 |  | -0.81 | 0.24 | -1.28 | -0.34 | <.001 |
| PVD | 0.41 | 0.28 | -0.13 | 0.96 | 0.14 |  | 0.26 | 0.16 | -0.05 | 0.57 | 0.10 |
| Stroke/TIA | 1.83 | 0.78 | 0.28 | 3.39 | <.05 |  | 1.06 | 0.33 | 0.42 | 1.71 | <.01 |
| COPD | -0.37 | 0.19 | -0.73 | 0.03 | 0.07 |  | -0.56 | 0.20 | -0.96 | -0.17 | <.01 |
| Hypertension | 0.13 | 0.20 | -0.28 | 0.53 | 0.54 |  | 0.28 | 0.12 | 0.04 | 0.51 | <.05 |
| Kidney disease |  |  |  |  |  |  |  |  |  |  |  |
| Stage 1 | Ref |  |  |  |  |  |  |  |  |  |  |
| Stage 2 | 0.13 | 0.29 | -0.45 | 0.71 | 0.66 |  | 0.15 | 0.14 | -0.12 | 0.41 | 0.28 |
| Stage 3 | -0.91 | 0.47 | -1.79 | -0.02 | <.05 |  | -0.40 | 0.29 | -0.98 | 0.17 | 0.17 |
| Stage 4 | -3.72 | 1.04 | -5.79 | -1.65 | <.001 |  | -2.77 | 0.77 | -4.29 | -1.25 | <.001 |
| Stage 5 | -3.01 | 2.25 | -7.47 | 1,45 | 0.18 |  | -3.46 | 1.92 | -7.28 | 0.36 | 0.07 |
| Diabetes | -0.10 | 0.15 | -0.40 | 0.20 | 0.50 |  | -0.29 | 0.10 | -0.50 | -0.09 | <.01 |
| Dementia | 1.11 | 1.34 | -0.02 | 3.76 | 0.41 |  | 1.62 | 1.08 | -0.53 | 3.77 | 0.14 |
| Liver disease | 0.44 | 2.35 | -0.42 | 5.11 | 0.85 |  | -0.90 | 0.82 | -2.52 | 0.72 | 0.27 |
| Cancer | 1.30 | 0.54 | 0.23 | 2.36 | <.05 |  | 0.58 | 0.42 | -0.25 | 1.42 | 0.17 |
| LV Function |  |  |  |  |  |  |  |  |  |  |  |
| ≥ 50% | Ref |  |  |  |  |  |  |  |  |  |  |
| 35-49% | -0.23 | 0.17 | -0.57 | 0.11 | 0.18 |  | -0.49 | 0.12 | -0.73 | -0.25 | <.001 |
| 20-34% | -0.47 | 0.65 | -1.39 | 0.46 | 0.32 |  | -0.53 | 0.20 | -0.93 | -0.13 | <.05 |
| <20% | 0.46 | 1.67 | -2.86 | 3.78 | 0.78 |  | -1.60 | 0.89 | -3.36 | 0.16 | 0.07 |
| Anemia | 1.28 | 0.32 | 0.64 | 1.91 | <.001 |  | -0.07 | 0.20 | -0.48 | 0.34 | 0.73 |
| Frailty | -0.73 | 0.05 | -0.83 | -0.63 | <.001 |  | -0.78 | 0.07 | -0.92 | -0.64 | <.001 |
| Major complications | -2.42 | 0.47 | -3.36 | -1.48 | <.001 |  | -2.23 | 0.48 | -3.17 | -1.29 | <.001 |

*AF atrial fibrillation; AV aortic valve; CAD coronary artery disease; Charlson CI Charlson comorbidity index; COPD chronic obstructive pulmonary disease; DAH days alive and out of hospital; LV left ventricular; MV mitral valve; PVD peripheral vascular disease; TV/PV pulmonic and tricuspid valve*
